# Immune Profile of the Normal Maternal-Fetal Interface in Rhesus Macaques and Its Alteration Following Zika Virus Infection

**DOI:** 10.3389/fimmu.2021.719810

**Published:** 2021-07-29

**Authors:** Matilda J. Moström, Elizabeth A. Scheef, Lesli M. Sprehe, Dawn Szeltner, Dollnovan Tran, Jon D. Hennebold, Victoria H. J. Roberts, Nicholas J. Maness, Marissa Fahlberg, Amitinder Kaur

**Affiliations:** ^1^Division of Immunology, Tulane National Primate Research Center, Covington, LA, United States; ^2^Department of Microbiology and Immunology, Tulane School of Medicine, New Orleans, LA, United States; ^3^Division of Reproductive and Developmental Sciences, Oregon National Primate Research Center, Beaverton, OR, United States; ^4^Division of Microbiology, Tulane National Primate Research Center, Covington, LA, United States

**Keywords:** fetal-maternal immunity, decidua, Zika, gammadelta T cells, dNK, decidual T cells, Treg, congenital viral infection

## Abstract

The maternal decidua is an immunologically complex environment that balances maintenance of immune tolerance to fetal paternal antigens with protection of the fetus against vertical transmission of maternal pathogens. To better understand host immune determinants of congenital infection at the maternal-fetal tissue interface, we performed a comparative analysis of innate and adaptive immune cell subsets in the peripheral blood and decidua of healthy rhesus macaque pregnancies across all trimesters of gestation and determined changes after Zika virus (ZIKV) infection. Using one 28-color and one 18-color polychromatic flow cytometry panel we simultaneously analyzed the frequency, phenotype, activation status and trafficking properties of αβ T, γδ T, iNKT, regulatory T (Treg), NK cells, B lymphocytes, monocytes, macrophages, and dendritic cells (DC). Decidual leukocytes showed a striking enrichment of activated effector memory and tissue-resident memory CD4+ and CD8+ T lymphocytes, CD4+ Tregs, CD56+ NK cells, CD14+CD16+ monocytes, CD206+ tissue-resident macrophages, and a paucity of B lymphocytes when compared to peripheral blood. t-distributed stochastic neighbor embedding (tSNE) revealed unique populations of decidual NK, T, DC and monocyte/macrophage subsets. Principal component analysis showed distinct spatial localization of decidual and circulating leukocytes contributed by NK and CD8+ T lymphocytes, and separation of decidua based on gestational age contributed by memory CD4+ and CD8+ T lymphocytes. Decidua from 10 ZIKV-infected dams obtained 16-56 days post infection at third (n=9) or second (n=1) trimester showed a significant reduction in frequency of activated, CXCR3+, and/or Granzyme B+ memory CD4+ and CD8+ T lymphocytes and γδ T compared to normal decidua. These data suggest that ZIKV induces local immunosuppression with reduced immune recruitment and impaired cytotoxicity. Our study adds to the immune characterization of the maternal-fetal interface in a translational nonhuman primate model of congenital infection and provides novel insight in to putative mechanisms of vertical transmission.

## Introduction

Successful maintenance of pregnancy requires a balance between sustaining an immune tolerant state to prevent rejection of foreign paternal-origin fetal antigens and at the same time protect the fetus against vertical transmission of microbial pathogens (1). The maternal-fetal interface is a rich and complex immune environment essential to fetal growth and survival that consists of maternal decidual tissue and fetal-origin cells such as placental trophoblasts, endothelial cells and Hofbauer cells (2, 3). The decidua contains a myriad of immune cells including antigen-presenting cells (DC and macrophages), stromal cells, NK cells, neutrophils, mast cells, regulatory T (Treg) cells, and adaptive T lymphocytes that are geared towards a dual role of establishing a tolerogenic environment conducive to placental embedding while maintaining local immunity against infections (1, 4, 5). Decidual leukocytes are phenotypically and functionally distinct from peripheral blood and change with gestational age. Most of the data in humans are from first trimester pregnancy with fewer studies in the second or third trimester (6–8). Studies in the first trimester of pregnancy in humans have shown that NK cells account for 50-90% of the decidual leukocyte population and predominantly have a CD56bright CD16– phenotype (7, 9). Despite the presence of cytolytic granules, decidual NK are poorly cytotoxic and differ from circulating NK cells in their functionality, expression of inhibitory and cytotoxicity receptors, and at the level of gene expression (7, 10–13). A similar dominant population of CD56+ NK cells has been described in rhesus macaque decidua (14–16). Along with decidual NK cells, Tregs and macrophages play a key role in maintaining an anti-inflammatory environment in the decidua (17, 18). Among adaptive immune subsets, CD3+ T lymphocytes constitute 5-20% of the decidual leukocyte population in early pregnancy and increase with gestational age; in contrast, B lymphocytes are rare (6, 19). Decidual T lymphocytes are enriched for differentiated CD8+ effector memory T cells that have reduced cytotoxic granule content and a transcriptional profile of exhaustion and activation genes distinct from circulating effector memory CD8+ T lymphocytes (20, 21). Decidual CD8+ T cells are also key players in recognition of foreign antigen and have been shown to contain fetal-specific CD8+ T cells (22–25). It is not known however whether decidual CD8+ T lymphocytes mount an antigen-specific response against pathogens; one study demonstrating decidual CD8+ T cells binding to EBV-specific tetramers was not conclusive (26, 27). Although the maternal-fetal interface has been extensively investigated for mechanisms underlying preterm birth and preeclampsia, relatively little is known about factors that prevent or allow maternal-fetal transmission of pathogens. We are using the rhesus macaque nonhuman primate (NHP) model to study factors predisposing to placental transmission of congenital infections such as cytomegalovirus (CMV) and Zika virus (ZIKV) (28–30).

Important similarities in placental biology make NHPs a suitable animal model to study vertical transmission of pathogens. Rhesus macaque placentation resembles humans in consisting of a hemochorial placenta with trophoblast invasion of the uterine wall (31, 32). However, macaque placentation is less deep with trophoblast invasion restricted to spiral arteries in the decidua and only occurring *via* the endovascular route (33). Macaque extravillous trophoblasts express the HLA-G ortholog, Mamu-AG, which has features of a nonclassical MHC class I molecule and was shown to be a ligand for the inhibitory killer immunoglobulin-like receptor Mamu-KIR3DL05 (34, 35). A recent transcriptomic analysis of human and macaque placenta revealed that the majority of human placental marker genes were shared between the two species, further validating the relevance of the NHP model (36). Of particular interest to the current study are the immune determinants at the maternal-fetal interface that protect against vertical transmission. A single-cell transcriptomics study of normal human pregnancy demonstrated the complexity of the immune populations in the maternal-fetal interface (37). The maternal-fetal interface of NHPs is less well delineated. Currently data are lacking on the decidual T lymphocyte phenotype, presence of nontraditional T cells, NK cell subsets, B cells, and myeloid cells in rhesus macaques across different gestational ages and their comparison to peripheral blood. The aim of this study was to conduct a comprehensive analysis of the immune composition of decidual and circulating leukocytes in normal rhesus macaque pregnancies throughout gestation and investigate changes in congenital infection. To this end we developed one 28-color and one 18-color flow cytometry panel to simultaneously analyze multiple innate and adaptive immune subsets in the decidua and blood of healthy rhesus macaques in the first, second and third trimester of pregnancy. We compared the frequency, phenotype, function and trafficking properties of decidual leukocytes with that of circulating leukocytes. In addition, we correlated changes in the decidual leukocyte composition with increasing gestational age. We then investigated changes following ZIKV infection in pregnancy by comparing normal decidua with decidual leukocytes and peripheral blood mononuclear cells from a previously reported study on ZIKV infection in pregnant rhesus macaques (29).

## Materials and Methods

### Animals and Study Design

Rhesus macaque dams of Indian ancestry from the Tulane National Primate Research Center (TNPRC) and the Oregon National Primate Research Center (ONPRC) were used for this study. All macaques were from the specific pathogen free (SPF) colonies of the respective primate centers. SPF macaques are CMV-seropositive but free of SIV, STLV, Type D retrovirus, and herpes B virus. At both institutions all animal procedures were performed according to approved Institutional Animal Care and Use Committee protocols. Peripheral blood (n=24) and decidua (n=11) from normal pregnant dams aged 3.6-17.0 years (mean=7.7 years, median=6.7 years) were collected for cross-sectional analysis of first, second, and third trimester gestation (**Table 1**). Information regarding timed-mating, housing, and mode of sampling are detailed in **Supplemental Table 1**. Decidua was obtained in 1^st^ trimester from day 44-50 gestation (n=3), 2^nd^ trimester at day 100 gestation (n=3), and 3^rd^ trimester from day 130-167 gestation (n=5) (**Supplemental Table 1**).

**Table 1:** Description of study cohort.

Trimester	1st Trimester		2nd Trimester		3rd Trimester	
Sample type	Decidua	PBMC	Decidua	PBMC	Decidua	PBMC
Uninfected (n)	3	6	3	11	5	7
Zika (n)	0	0	1	1	9	8

Blood and decidua were also collected close to or at the time of Caesarian section (C-section) from a cohort of ZIKV-infected dams (n=10) enrolled in a separate previously published study (29). The group consisted of pregnant Indian-ancestry rhesus macaques inoculated subcutaneously with a single dose of 1x10^4^ PFU ZIKV Rio-U1 at the first (n=1), second (n=3), or third trimester (n=6) gestation followed by C-section and decidua collection at 2^nd^ or 3^rd^ trimester gestation (**Table 1** and **Supplemental Table 2**). Peripheral blood was obtained 5-41 days post ZIKV infection while placenta was collected at the time of study C-section or spontaneous abortion which ranged from 16-56 days post ZIKV infection (**Supplemental Table 2**). One decidua was collected in the second trimester at 64 days gestation age while the remaining 9 decidua samples were collected in the third trimester at 142 to 157 days gestation age (**Table 1** and **Supplemental Table 2**).

### Isolation of Leukocytes From Blood and Decidual Tissue

Peripheral blood mononuclear cells (PBMC) were isolated from blood collected in anticoagulant EDTA or heparin tubes by density gradient centrifugation with Lymphocyte Separation Medium (LSM; MP Biomedicals) or Lymphoprep (Stemcell Technologies). Isolated PBMC were cryopreserved using serum-free Bambanker cryopreservation media (Bulldog Bio) and stored in liquid nitrogen until use.

Following C-section, decidua from ONPRC study animals was dissected from the maternal side of the placenta and shipped overnight to TNPRC in R10 media (RPMI supplemented with Hepes, L-glutamine, and 10%FBS) on ice for decidual leukocyte isolation. Placental samples collected at TNPRC were immediately processed for decidual leukocyte isolation. Blood clots were removed from the maternal side of the placenta and decidual tissue carefully dissected away from the placenta. Visible blood vessels were removed and decidual tissue pieces were then washed extensively with sodium chloride 0.9% (Hanna Pharmaceutical Supply Co.) to remove any debris and remaining blood. Clean decidua was dissected into 2 mm^2^ pieces and subjected to three rounds of digestion by shaking at 60 RPM at 37°C for 30 minutes in digestion media containing 0.1mg/mL DNAse I (Millipore Sigma 11284932001) and collagenase IV 1 mg/mL (Millipore Sigma C5138-100MG). Released cells in solution were collected in between each digestion and washed. After the last digestion, residual cells were passed repeatedly through a sterile 18-gauge animal feeding needle (Fisherbrand™) for gentle mechanical sheering before straining through sterile 70μm filters. Cells from each digestion were pooled. The resulting single cell suspension was layered over a density gradient LSM (MP Biomedicals) and spun at 1500 rpm for 45 minutes with no brakes. Lymphocytes were carefully collected from the top layer, washed, and cryopreserved in DMSO supplemented with 90% FBS or in serum-free Bambanker cryopreservation medium (Bulldog Bio).

### Phenotyping by Multicolor Flow Cytometry

Two flow cytometry panels were used to phenotype the leukocytes, one adaptive 28-color panel, and one innate 18-color panel (**Supplemental Table 3**). In the same batch experiment, decidual leukocytes and PBMC from normal and ZIKV-infected animals were gently thawed using standard protocols supplemented with 17.5µg/mL DNAse I (Millipore Sigma 11284932001). 1-3 million live lymphocytes were used for staining. Briefly, cells were stained with live/dead discriminating dye for 20min at room temperature (RT). Cell suspensions were washed with 2% FBS/PBS buffer and the 28-color panel was stained sequentially with anti-Vα24-PE and BV421-CD1d tetramer first (20 minutes incubation at RT), followed by addition of the chemokine receptor antibodies anti-CCR5-BUV737 and anti-CXCR3-PE-Cy7 (20 minutes incubation at 37°C), and finally by addition of the chemokine receptor antibodies anti-CCR4-BV510 and anti-CCR6-BV650 (20 minutes incubation at 37°C) with no washes in between. Last, the remaining surface cocktail was added and the tubes incubated for 20 minutes at RT with antibodies outlined in **Supplemental Table 3**. Cells were then washed with 2% FBS/PBS prior to incubating with BD Cytofix/Cytoperm™ solution (BD Biosciences) for 20 minutes at RT followed by washing with BD Perm/Wash™ buffer (BD Biosciences). Intracellular antibodies were then added for a 20-minute incubation at RT. The 18-color panel was stained using the same applicable steps. Cells were fixed with Stabilizing Fixative (BD Biosciences; Cat# 338036) and acquired the next day on the BD FACSymphony A5 and BD LSRFortessa X-20 using BD FACSDiva version 9.1 and 9.0 respectively. A mean of 244,000 (50,000 to 718,000) Time/CD45+/Live events were collected. FMO controls included in the experiments were BB700-PD-1, BV650-CCR6, PE-Cy7-CXCR3, AL647-CX3CR1, PE-Vα24, and APC-NKG2D. Single color controls were acquired in all experiments and compensation and analysis was performed in FlowJo software v10.7 (BD Biosciences).

### Data Analysis

Flow cytometry data was analyzed using FlowJo v10.7 (BD Biosciences). Statistical analysis was performed using GraphPad Prism version 9.0.1 (GraphPad Software Inc.) and R (CRAN). tSNEs were calculated in FlowJo using the default settings for opt-SNE. An equal number of cells per tissue were used when analyzing PBMC *versus* decidua in uninfected animals. An equal number of cells per condition were used when analyzing ZIKV-infection *versus* uninfected decidua. Boxplots and tSNE renderings were created using the tidyverse and ggpubr packages in R. Data throughout the result section are reported as mean ± standard deviation (SD) unless noted.

## Results

### Circulating and Decidual Leukocyte Composition in Normal Pregnancy

**Figure 1:**
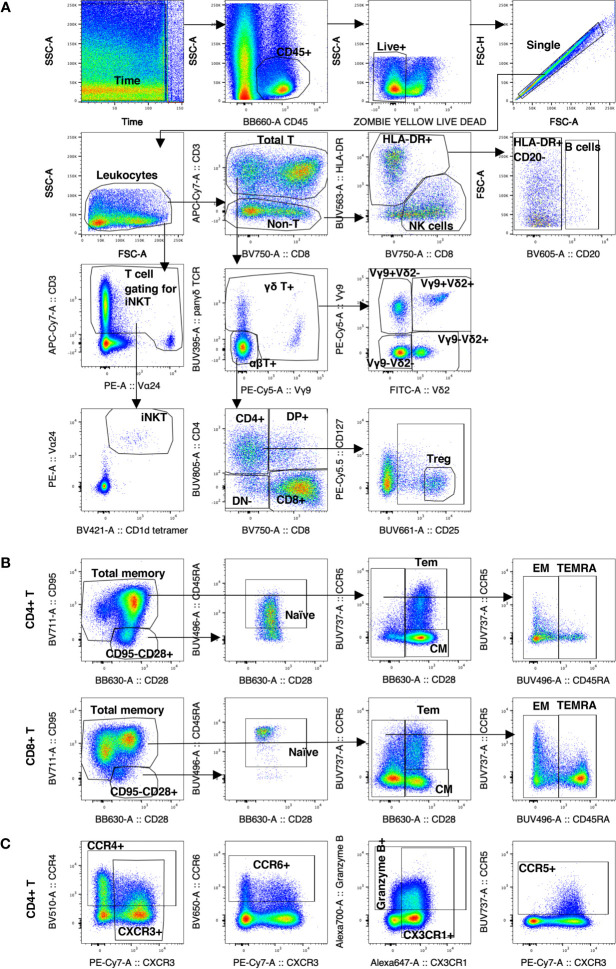
Gating strategy of decidual leukocytes and PBMC in normal rhesus macaques. **(A)** Gating strategy in 28-color panel to identify αβ T cells, NK cells, B cells, myeloid cells (HLA-DR+ CD20-), iNKTs, γδ T cells, Tregs. **(B)** Gating strategy in CD4+ T and CD8+ T to delineate the naïve memory T cells subsets of CM, Tem, EM, and TEMRA. **(C)** Gating strategy of chemokine receptor expression.

**Figure 2:**
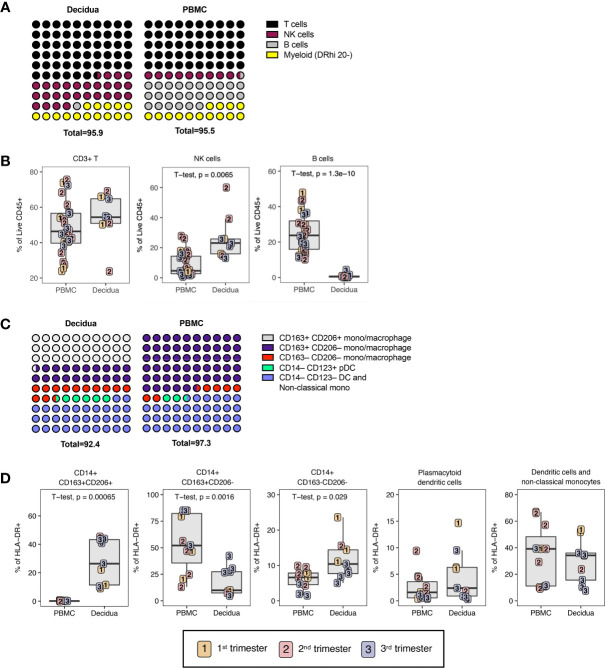
Leukocyte composition of PBMC and decidua in normal rhesus macaques. **(A)** Comparison of relative proportions of the CD45+ leukocytes in decidua and PBMC. **(B)** Frequency of total T lymphocytes, NK cells, and B lymphocytes in PBMC and decidua during the first (yellow), second (red) and third trimester (blue) of pregnancy in normal rhesus macaques. **(C)** Comparison of relative proportions of the myeloid cells (CD3-CD20-HLA-DR+) in decidua and PBMC. **(D)** Frequency of myeloid cells (CD3-CD20-HLA-DR+) in PBMC and decidua of monocyte/macrophages subsets, and dendritic cells (DC). P-values <0.05 (unpaired t-test) shown on the plots.

To examine immune cell populations at the maternal-fetal interface in normal rhesus macaques, we conducted a cross-sectional analysis of first (n=3), second (n=3), and third (n=5) trimester decidua obtained at the time of C-section in healthy pregnancy and compared it with blood obtained from gestation-matched healthy, uninfected dams experiencing normal pregnancies (**Table 1**). PBMC and decidual leukocytes were evaluated with two flow cytometry panels, one 28-color panel focused on adaptive immunity, and one 18-color innate cell focused panel (**Supplemental Table 3**). T lymphocytes, NK cells, B lymphocytes, and cells of the myeloid lineage were enumerated using the gating strategy in **Figures 1A–C**. Definitions used in this study for each analyzed cell type in the two flow panels were based on markers in NHPs that are equal to their human counterparts (**Table 2**). For example, NK cells which are CD3– CD16^bright^ CD56^dim^ or CD16^–/dim^ CD56^bright^ in humans are defined as CD3– HLA-DR^lo/–^ CD8+ in rhesus macaques (38). T lymphocytes were the predominant leukocytes in both PBMC (mean ± SD; 48.0 ± 14.7%) and decidua (54.2 ± 12.6%) but not significantly different between the two compartments (**Figures 2A, B**). NK cells were significantly increased in the decidua with ~3-fold greater frequency compared to PBMC, and were highest in the 2^nd^ trimester (**Figures 2A, B**). No relationship between NK cells and gestational age was found in the decidua (**Supplemental Figure 1**). B lymphocytes were close to absent in decidual tissue in contrast to PBMC where they made up 25.3 ± 10.8% of the live CD45+ leukocytes (**Figures 2A, B**). In the myeloid antigen presenting compartment, which was defined as CD3– CD20– CD8^lo/-^HLA-DR+ (**Table 2**), a population of CD14+ tissue macrophages expressing both CD163+ and CD206+ were found in the decidua (**Figures 2C, D**). This population was virtually absent in PBMC (0.1 ± 1.2%) but represented 28.1 ± 14.7% of HLA-DR+ leukocytes in the decidua (**Figure 2D**). CD14+ CD163+ CD206– myeloid cells were found in PBMC and decidua at 54.1 ± 25.8% and 18.3 ± 12.8% respectively of myeloid HLA-DR+ expressing cells (**Figure 2D**). These cells are a mix of classical and intermediate monocytes in the PBMC and recently infiltrated monocytes and macrophages in tissues (39, 40). CD14+ CD163– CD206– cells were significantly increased in the decidua compared to PBMC (**Figure 2D**). Plasmacytoid DC (pDC) frequency ranged between 0.3 to 14.7% of HLA-DR-expressing cells in decidua but were not significantly different from PBMC (**Figure 2D**). CD14– CD123– HLA-DR+ leukocytes, which can be either conventional DCs or CD14– CD16+ non-classical monocytes in circulation, were similar between PBMC and decidua (34.4 ± 21.3% and 29.8 ± 15.8% respectively of HLA-DR-expressing cells).

**Table 2:** Flow cytometric definition of cell types.

Cell type*	Definition	Panel
αβ T cells	CD3^+^ panγδ^–^	Adaptive 28-color
	CD4+: CD4^+^ CD8^–^	
	CD8+: CD4^–^ CD8^+^	
	Double Positive (DP+): CD4+ CD8+	
	Double Negative (DN–): CD4– CD8–	
γδ T cells	CD3+ panγδ+	Adaptive 28-color
	Vγ9+ Vδ2+	
	Vγ9+ Vδ2–	
	Vγ9– Vδ2+	
	Vγ9– Vδ2–	
iNKT	CD3+ Vα24+ CD1d tetramer+	Adaptive 28-color
T regulatory cells	CD3+ CD4+ CD127– CD25^hi^	Adaptive 28-color
Memory T cells	Naïve: CD95– CD28+ CD45RA+	Adaptive 28-color
In CD4+ αβ T	Total Memory: CD95+ CD28+/–	
In CD8+ αβ T	Central Memory (CM): CD95+ CD28+ CCR5–	
In γδ T	Transitional Effector Memory (Tem): CD95+ CD28+ CCR5+	
	Effector Memory (EM): CD95+ CD28– CD45RA–	
	Terminal Effector Memory (TEMRA): CD95+ CD28– CD45RA+	
	Tissue Resident Memory (Trm): CD95+ CD28+/– CD103+ CD69+	
T helper subsets	CD3+ CD4+	Adaptive 28-color
	CXCR3+ CCR5+/– Th1-like	
	CCR4+ Th2-like	
	CCR6+ Th17-like	
NK cells	CD3– HLA-DR^lo/-^ CD8+	Innate 18-color and adaptive 28-color
	CD16+ CD56–	
	CD16– CD56+	
	CD16+ CD56+	
	CD16– CD56–	
B cells	CD3– HLA-DR^hi^ CD20+	Adaptive 28-color
Macrophages in Decidua	CD3– CD20– HLA-DR+ CD8– CD14+	Innate 18-color
	CD163+ CD206+	
	CD163+ CD206–	
	CD163– CD206–	
Monocytes in PBMC	CD3– CD20– HLA-DR+ CD8–	Innate 18-color
	Classical monocytes: CD14+ CD16–	
	Intermediate monocytes: CD14+ CD16+	
	Non-classical monocytes and DCs: CD14– CD16+	
Dendritic cells (DC)	CD3– CD20– HLA-DR+ CD14–	Innate 18-color
	Conventional DC, myeloid DC, and non-classical monocytes: CD123–	
	Plasmacytoid DC: CD123+	

*Cells are pre-gated as Live+ Single CD45+ Leukocytes.

**Figure 3:**
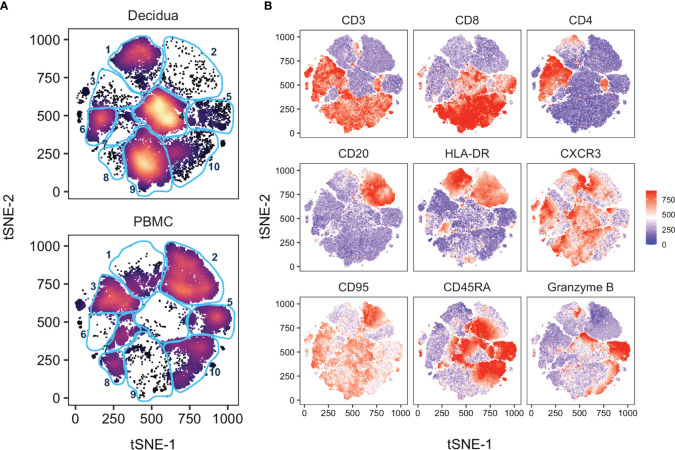
tSNE analysis of PBMC and decidua in normal rhesus macaques. **(A)** tSNE plot representing an equal number of CD45+/live/single cell/leukocytes from decidua (n = 9) and PBMC (n = 24) of normal pregnant rhesus macaque samples. The 28-color adaptive panel was used to generate this plot. Blue gates were manually drawn based on clustering patterns. **(B)** Individual MFI gradients of nine markers on the tSNE map. Red coloring represents high MFI and blue coloring represents low MFI. Remaining gradients are found in **Supplemental Figure 2**.

To further probe differences and assess global changes of PBMC *vs*. decidua that may have been missed during manual gating, we performed an unbiased clustering analysis by t-distributed stochastic neighbor embedding (tSNE) using CD45+ live+ single cells in the 28-color panel focused on T, NK, and B cells. There were clear differences in leukocyte populations between sample types (**Figure 3**). Gradients colored by MFI depicted distinct CD3+ CD4+ (clusters 3 and 6) and CD3+ CD8+ T cells (clusters 8, 9, and 10) between the circulation and the decidual T cells (**Figure 3**). The near absence of B cells in decidua observed by manual gating was confirmed by the cluster of HLA-DR+ CD20+ (cluster 2) events only found in PBMC (**Figure 3**). CD95/Fas receptor, a marker of T cell memory in NHPs, was found in cluster 9, representing CD8+ T cells, which was predominantly found in the decidua. Granzyme B was found in both tissues but in different clusters which represented unique types of NK cells (**Figure 3**). An additional 17 more markers used to perform the tSNE algorithm are found in **Supplemental Figure 2**.

### Classical and Unconventional T Lymphocytes Distribution in PBMC and Decidua

**Figure 4:**
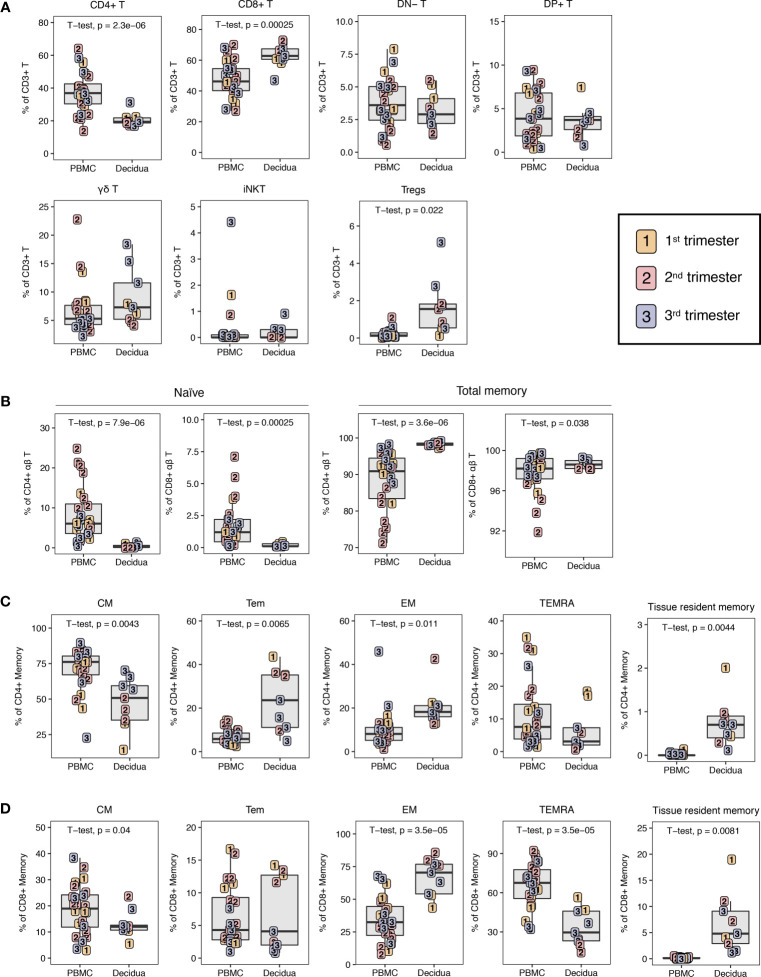
T lymphocyte composition in PBMC and decidua from first to third trimester in normal pregnant rhesus macaques. **(A)** Frequency of conventional and innate T cells in first (yellow), second (red) and third trimester (blue) of pregnancy in PBMC and decidua of normal rhesus macaques. **(B)** CD4+ T and CD8+ T naive and total memory. **(C)** CD4+ T memory subsets. **(D)** CD8+ T memory subsets. P-values <0.05 (unpaired t-test) shown on the plots.

αβ T and γδ T cells were distinguished by expression of the pan-γδ T cell marker on total CD3+ T lymphocytes (**Figure 1A**). In classical αβ T lymphocytes, the CD4+ T cells were reduced in decidua compared to PBMC from 37.4 ± 12.0% to 20.7 ± 4.1% (**Figure 4A**). Consistent with human data, CD8+ T cells were the dominant T lymphocyte population in macaque decidua and present at significantly higher frequencies (63.1 ± 7.4%) compared to PBMC (47.5 ± 11.6%) (6, 19). No significant differences were found in double-positive (DP) or double-negative (DN) T cells (**Figure 4A**). In the non-classical T lymphocyte subset, γδ T cells were increased in decidua compared to PBMC but did not reach statistical significance unless only third trimester animals were compared (two-tailed unpaired t-test, p-value = 0.0008) (**Figure 4A**).

Among unconventional T lymphocytes, we evaluated invariant natural killer T (iNKT) cells. iNKT are an innate subset of T lymphocytes that respond to glycolipid antigens presented on CD1d molecules. These cells have been reported in human decidua but there are no data in NHPs (41, 42). Here, we sought to identify their presence in rhesus macaque decidua using the stringent identifying criteria of Vα24 TCR-expressing T lymphocytes binding to α-galactoslyceramide (αGC)-loaded CD1d tetramers (41–44). A detectable but sparse population of iNKTs with frequencies ranging between 0.036 to 0.918% of CD3+ T lymphocytes were detected in 6 of 9 decidua samples and at frequencies ranging between 0.005 to 4.4% in 17 out of 24 PBMC samples. We did not detect any difference in iNKT frequency between PBMC and decidua (**Figure 4A**).

In agreement with data from humans (6, 19), the decidua had a significantly increased frequency of CD4+ T regulatory cells (Tregs) when compared to PBMC (**Figure 4A**). In this study, CD4+ Tregs were defined based on low CD127 expression on CD25hi CD4+ T lymphocytes, a population that has previously been shown in both humans and macaques to be FoxP3-positive and display Treg functionality of suppressive activity (45). Tregs accounted for 7.9 ± 5.5% of decidual CD4+ T lymphocytes while they were present in only 0.5 ± 0.4% of circulating CD4+ T lymphocytes. If assessed as a fraction of T cells, Tregs were 1.7 ± 1.4% in decidua and 0.2 ± 0.2% in PBMC. Decidual Tregs increased with gestation as a positive correlation (R^2^ = 0.5392, p-value = 0.0243) between Treg frequency and days of gestation was detected (**Supplemental Figure 1**).

### Memory CD4+ and CD8+ T Lymphocytes in PBMC and Decidua

Using a combination of CD95, CD28, CCR5, CD45RA, CD69, and CD103 we delineated naïve (CD95– CD28+ CD45RA+) and five populations of CD95+ memory CD4+ T or CD8+ T lymphocytes (**Table 2** and **Figure 1B**). Memory subsets included CD95+ CD28+ CCR5–CD45RA– central memory (CM), CD95+ CD28– CCR5+/– CD45RA– effector memory (EM), CD95+ CD28+ CCR5+ CD45RA–transitional effector memory (Tem), CD95+ CD28– CCR5+/– CD45RA+ terminally differentiated effector memory (TEMRA), and CD95+ CD69+ CD103+ tissue-resident memory (Trm).

The decidua exhibited a distinct phenotype of memory cells and a near absence of naïve cells (**Figure 4B**). The majority of CD4+ T memory lymphocytes in decidua and PBMC were CM but PBMC had an increased frequency of CM CD4+ T compared to decidua, whereas decidua had higher frequencies of EM and Tem CD4+ T lymphocytes (**Figure 4C**). CD4+ Trm were present at a small frequency of 0.7 ± 0.5% of CD4+ memory while no cells of this phenotype were found in PBMC (**Figure 4C**). Effector memory (EM) constituted the predominant subset of CD8+ T memory lymphocytes in decidua at a frequency of 67.0 ± 13.4% that was significantly higher than PBMC. Conversely, in PBMC, the majority of CD8+ memory T cells showed a terminally differentiated TEMRA phenotype of 65.6 ± 16.7%. The decidua had a reduced frequency of CD8+ T CM at 13.1 ± 4.9% compared to 18.7 ± 9.4% in the PBMC, which is similar to what was seen in the CD4+ T lymphocyte memory compartment (**Figure 4D**). Decidua also had a clear population of CD8+ Trm cells (6.8 ± 5.4%), which was completely absent in the PBMC (**Figure 4D**).

**Figure 5:**
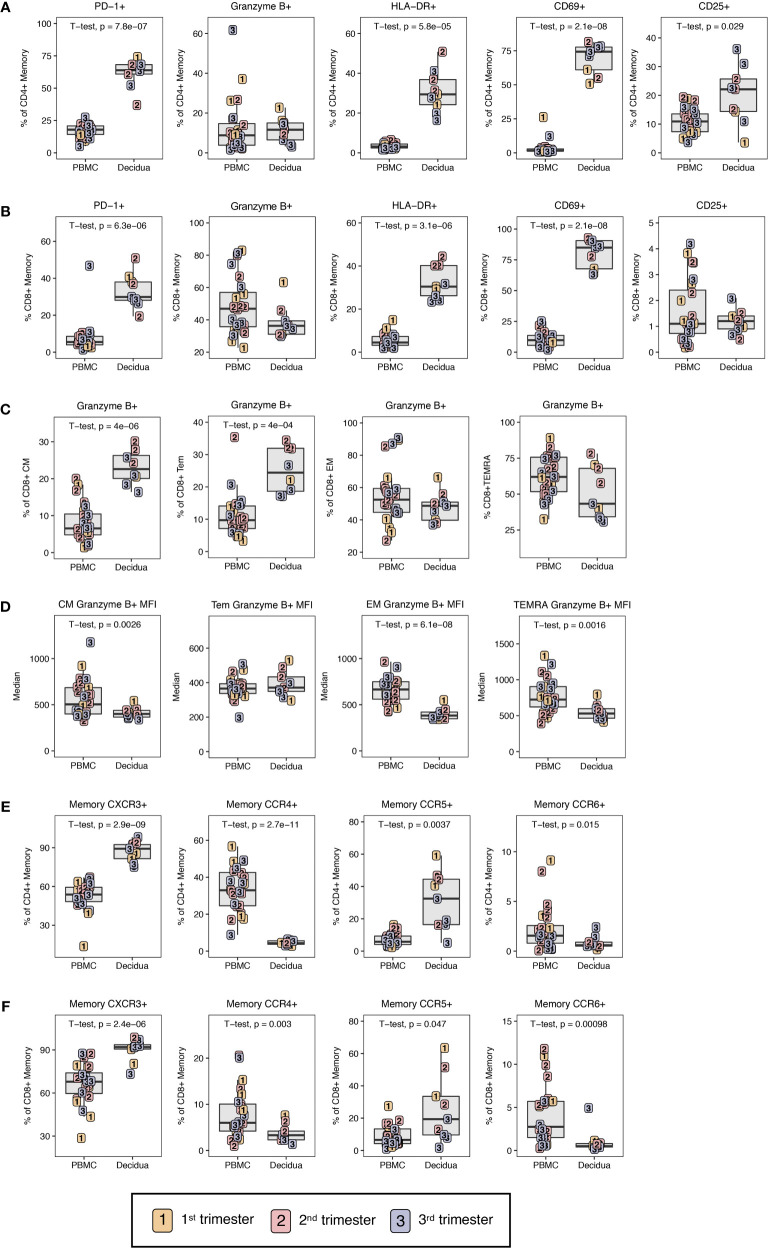
Phenotypic characterization of T lymphocyte memory. **(A)** Phenotype of CD4+ T cell and **(B)** CD8+ T cell memory by PD-1, Granzyme B, HLA-DR, CD69, and CD25 in first (yellow), second (red) and third trimester (blue) of pregnancy in PBMC and decidua of normal rhesus macaques. **(C)** Cytotoxic potential in CD8+ T memory subsets by Granzyme B frequency in CM, Tem, EM, and TEMRA. **(D)** MFI of Granzyme B in CD8+ T memory subsets. **(E)** Chemokine receptor expression on memory CD4+ and **(F)** CD8+ T cells. P-values < 0.05 (unpaired t-test) shown on the plots.

To evaluate the functional potential of circulatory T cells to those at the maternal-fetal interface, markers related to exhaustion, cytotoxicity, and activation were investigated using the gating strategy shown in **Supplemental Figure 3**. The exhaustion marker PD-1 was significantly elevated in decidual CD4+ and CD8+ T lymphocytes compared to PBMC with roughly 4-fold higher frequencies of PD-1+ CD4+ or CD8+ T lymphocytes in the decidua (**Figures 5A, B**). This is similar to what has been observed for effector memory T lymphocytes in humans between PBMC and decidual T lymphocytes (25). Despite high expression levels of PD-1, memory CD4+ and CD8+ T lymphocytes in the decidua were highly activated with significantly increased frequencies of CD69+ and HLA-DR+ cells as compared to PBMC (**Figures 5A, B**). CD25, a marker of activation, was significantly increased on CD4+ T lymphocytes in the decidua compared to PBMC (**Figure 5A**).

To investigate cytotoxic potential, we examined granzyme B expression in memory CD4+ and CD8+ memory T lymphocytes. Although decidual CD8+ memory T cells as a whole had lower frequencies of granzyme B-positive cells compared to PBMC (38.6 ± 9.9 *versus* 48.4 ± 17.4), these differences did not reach statistical significance (**Figure 5B**). However, evaluation of memory subsets yielded clear differences between PBMC and decidua. Among circulating CD8+ memory T lymphocytes, TEMRA contained the highest frequency of granzyme B-positive cells (62.0 ± 15.9) followed by EM at 55.5 ± 17.2%, and Tem and CM at <50% (**Figure 5C**). Comparison between the two compartments showed significantly higher frequency of granzyme B-positive Tem and CM CD8+ T lymphocytes in the decidua compared to PBMC (**Figure 5C**). No difference was found in CD8+ EM or TEMRA. To further assess granzyme B expression in decidual and PBMC CD8+ T lymphocytes, we measured the median fluorescence intensity (MFI) of granzyme B in the different CD8+ T lymphocyte memory subsets as an approximation of granzyme B content. Across the memory subsets of TEMRA, EM and CM, granzyme B MFI in decidual leukocytes was significantly lower compared to their circulating counterparts (**Figure 5D**). Therefore, despite similar frequencies of granzyme B-positive cells, the predominant decidual subsets of EM and TEMRA CD8+ T memory have reduced granzyme B content and are potentially less cytotoxic compared to the corresponding memory subsets in circulation. It is noteworthy that the granzyme B content as measured by MFI was significantly lower in decidual CM compared to PBMC CM despite the frequency of granzyme B-positive CM CD8+ T lymphocytes being significantly higher in decidua (**Figures 5C, D**).

In memory T cells, the trafficking phenotype was also investigated by Th-subset associated receptors CXCR3, CCR6, CCR5, and CCR4 (**Figures 1C**, **5E, F**). Consistent with previous reports in humans (25), decidual memory CD4+ T lymphocytes have an increased frequency of CXCR3 expression in contrast to the peripheral leukocytes from 87.3 ± 7.5% in decidua to 52.7 ± 10.8% in PBMC indicating Th1 skewing (**Figure 5E**). The same is true for CD8+ T lymphocytes where 90.0 ± 7.6% expressed CXCR3 and only 66.2 ± 13.6% did so in PBMC (**Figure 5F**). CCR5 was also increased in decidual CD4+ and CD8+ T lymphocytes compared to PBMC. Little to no CCR4 and CCR6 expression was found in the decidua, which was significantly different from PBMC where these chemokines were well-expressed (**Figures 5E, F**).

### γδ T Lymphocytes in PBMC and Decidua

Next, the non-classical T lymphocytes were investigated. There are limited data on γδ T at the maternal-fetal interface. In one study in humans, γδ T cells were shown to be increased in the decidua in contrast to peripheral blood while having a reduced Vδ2 frequency (46). We investigated the frequency, CD4/CD8 phenotype, memory phenotype, and activation status of total γδ T lymphocytes detected by the pan- γδ TCR antibody. We also evaluated γδ T subsets based on TCR chain usage of total γδ T by using NHP cross-reactive antibodies against the Vγ9 TCR and Vδ2 TCR chain.

**Figure 6:**
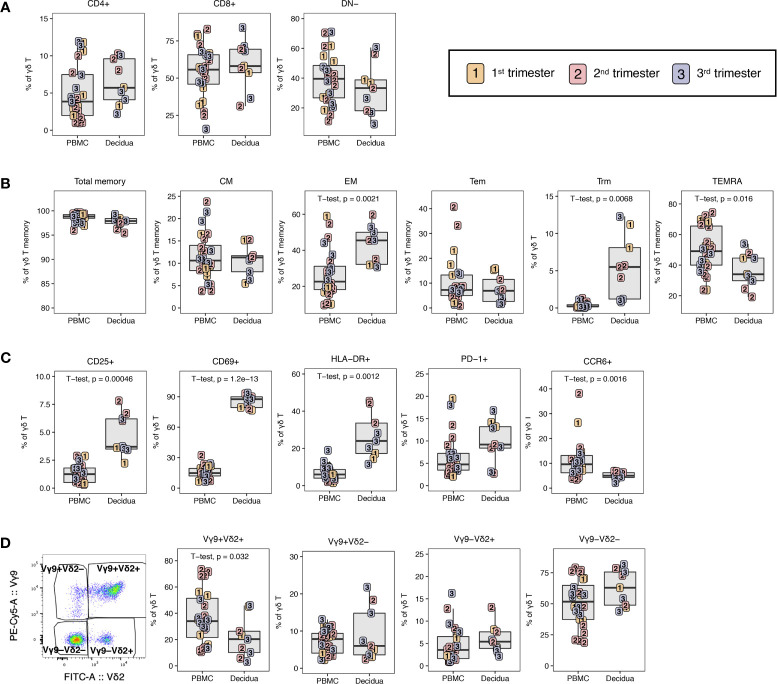
γδ T lymphocytes in PBMC and decidua of normal rhesus macaque pregnancies. **(A)** CD4 and CD8 expression on γδ T lymphocytes in PBMC and decidua in first (yellow), second (red) and third trimester (blue) of pregnancy of normal rhesus macaques. **(B)** Memory phenotype of γδ T lymphocytes. **(C)** Phenotype of activation, exhaustion, cytotoxic potential and chemokine receptor expression in γδ T lymphocytes. **(D)** Representative flow plot of Vγ9 and Vδ2 chains and the Vγ9 and Vδ2 subsets in PBMC and decidua. P-values < 0.05 (unpaired t-test) shown on the plots.

The majority of both decidual and peripheral blood γδ T lymphocytes were CD8+ or DN for CD4 and CD8 (**Figure 6A**). They were nearly entirely of a memory phenotype in both PBMC and decidua; however, CD8+ EM predominated in the decidua (42.6 ± 10.0) and TEMRA were most abundant in the PBMC (49.9 ± 14.8%) (**Figure 6B**). As expected, tissue resident Trm were detected in the decidua (5.5 ± 4.1%) but not in PBMC (**Figure 6B**). Similar to what was observed in αβ T lymphocytes, total γδ T lymphocytes appeared to be more activated in the decidua. Expression of CD25, CD69 and HLA-DR were significantly increased compared to PBMC, and this was accompanied with a concurrent increase of PD-1 expression in the decidua (**Figure 6C**). Like the αβ T lymphocytes, there was a significant reduction in the frequency of CCR6-expressing cells in the decidual γδ T cells (**Figure 6C**).

Using the individual gamma and delta TCR chains, four subsets of γδ T cells could be distinguished. The double positive, Vγ9+ Vδ2+ was present at significantly lower frequencies in the decidua compared to PBMC (**Figure 6D**). The single positive Vγ9+ Vδ2– and Vγ9– Vδ2+ γδ T were minor populations in both PBMC and decidua ranging in frequency from 1.0 to 21.8% of γδ T cells (**Figure 6D**). The innate-like double negative Vγ9– Vδ2– subset was increased in the decidua, although not significantly (**Figure 6D**).

### Global Analysis of Innate Immune Cells in Decidua and PBMC

**Figure 7:**
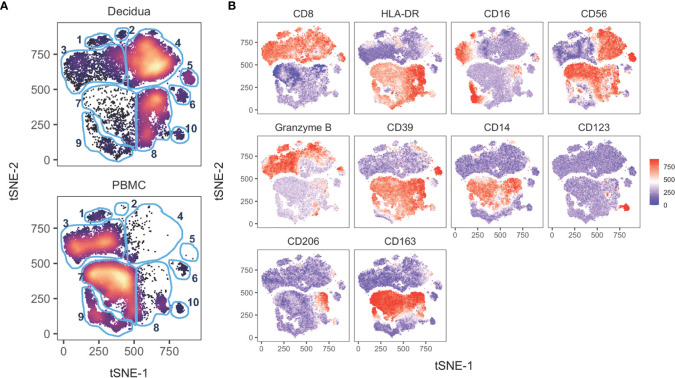
tSNE of 14 innate cell markers of PBMC and decidual leukocytes in normal pregnant rhesus macaques. **(A)** tSNE plot representing an equal number of CD45+/live/single cells/CD3-CD20- cells from decidua (n = 8) and PBMC (n = 11) of rhesus macaques experiencing a normal pregnancy. The 18-color innate panel was used to generate this plot. Blue gates were manually drawn based on clustering patterns. **(B)** Individual MFI gradients of ten markers on the tSNE map. Red coloring represents high MFI and blue coloring represents low MFI. Remaining gradients are found in **Supplemental Figure 4**.

To analyze global differences of innate leukocytes of the peripheral blood compared to decidua, we performed a tSNE analysis on the 18-color innate cell panel. Only live CD45+ CD3– CD20–cells were included in the computation. Stark differences could be observed between the PBMC and the decidual cells (**Figure 7A**). The top half of the tSNE shows clusters 1,2,3,4,5 which consist of NK cells based on their CD8+ and HLA-DRlo/– expression (**Figures 7A, B**). In decidua, cluster 4 was a dominant cluster of CD16– CD56+ NK cells which was absent in the PBMC tSNE. On the other hand, peripheral blood NK cells like those present in cluster 3 exhibited a CD16+ CD56– or CD16–CD56– phenotype (**Figures 7A, B**). The lower half of the tSNE expresses HLA-DR+ which by the removal of CD20+ cells in the gating preceding tSNE analysis is consistent with myeloid antigen-presenting cells (APCs). Cluster 8 is the major myeloid APC subset in the decidua which is absent in PBMC and is likely mostly comprised of monocytes or macrophages based on CD14 and CD163 expression (**Figures 7A, B**). In the PBMC, clusters 7 and 9 are predominant subsets which are rare in the decidua. Based on gradient expression of CD14, CD16, and CD163, cluster 7 is likely classical monocytes based on CD14+ CD16– CD163+ events, while cluster 9 is consistent with intermediate, non-classical monocytes, or conventional DCs (**Figures 7A, B**). Cluster 10 appears to be a pDC population based on CD123 expression. Four additional markers used to perform the tSNE clustering algorithm are shown in **Supplemental Figure 4**.

### NK Cells in PBMC and Decidua

**Figure 8:**
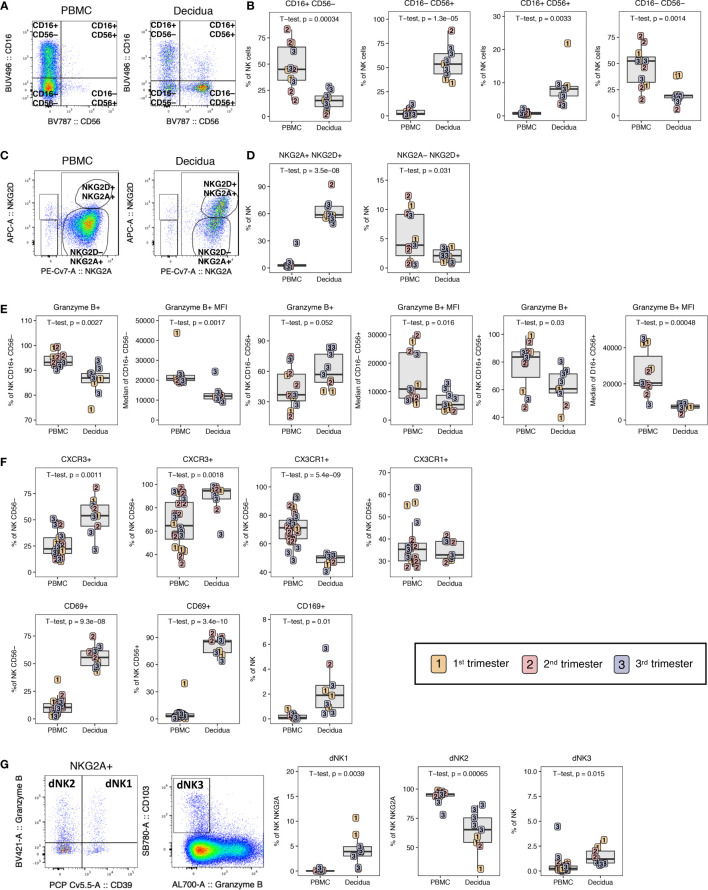
NK cell composition in PBMC and decidua of normal rhesus macaque pregnancies. **(A)** Representative flow plots of NK cells CD16/CD56 subsets. **(B)** NK cell subset frequencies in PBMC and decidua first (yellow), second (red) and third trimester (blue) of normal pregnant rhesus macaques. **(C)** NKG2A/NKG2D flow plots with representative gating of PBMC and decidua. **(D)** Frequency of NKG2A+NKG2D+ and NKG2A-NKG2D+ NK cells in PBMC and decidua. **(E)** Granzyme B expression frequency and MFI on NK cell subsets. **(F)** CD56- *vs* CD56+ NK cell expression of CXCR3, CX3CR1, CD69 and the CD169 on total NK cells. **(G)** Representative flow plot of dNK1, dNK2, and dNK3 subsets with box plots showing their frequency in PBMC and decidua. P-values < 0.05 (unpaired t-test) shown on the plots.

The decidual tissue has been reported to be enriched with innate cells such as NK cells in previous non-human primate studies (15). Here, we sought to complement these data by identifying the activation status of innate cells and their unique phenotypes. A distinct phenotype of CD16– CD56+ single-positive NK cells was found to predominate in the decidua (55.3 ± 16.1%), in stark contrast to PBMC where the major NK cell subset identified were CD16+ CD56– cells (48.2 ± 20.2%) (**Figures 8A, B**). Interestingly, a distinct CD16+ CD56+ population of NK cells was found in the decidual tissue (8.4 ± 5.2%) which was virtually absent in PBMC (**Figure 8B**). These have been described in human decidua as CD56bright and CD16+/dim NK cells (9). In addition, double-negative CD16– CD56– NK cells were significantly increased in the PBMC compared to decidua (**Figure 8B**). NKG2A and NKG2D dual positivity also defined a subset of NK cells found almost exclusively in the decidua (62.9 ± 12.1%) (**Figures 8C, D**). NKG2D+ NKG2A– cells were greatly reduced in decidual NK cells as compared to peripheral NK cells (**Figures 8C, D**).

To analyze the cytotoxic capacity of these cells, we measured the frequency of granzyme B+ cells as well as the MFI, a proxy of intracellular granzyme B content. The frequency and MFI of granzyme B in the cytotoxic CD16+ CD56– and the double-positive CD16+ CD56+ NK subsets were significantly lower in the decidua compared to PBMC (**Figure 8E**). Interestingly, the frequency of granzyme B-positive CD16– CD56+ NK cells was significantly higher in the decidua compared to PBMC; however, the granzyme B MFI of this decidual subset was significantly lower as was true of the other decidual NK subsets (**Figure 8E**). These data show that the dominant decidual CD16– CD56+ NK subset contain high proportions of cytotoxic NK, albeit with less cytotoxicity. It is noteworthy that the frequency of the granzyme B-positive cells in all the NK subsets was highest in third trimester decidua (**Figure 8E**).

Similar to T lymphocytes, decidual NK cells expressed more CXCR3 in both CD56– and CD56+ subsets compared to PBMC (**Figure 8F**). We also evaluated expression of CX3CR1, a chemokine receptor which is upregulated in cells that home to sites experiencing endothelial inflammation and is elevated on a subset of memory CD8+ T lymphocytes and myeloid cells (47). We found decreased expression of CX3CR1 on CD56– NK cells of the decidua (47.8 ± 4.6) compared to PBMC (71.1 ± 11.1) but not in CD56+ NK cells where similar expression levels were found (**Figure 8F**). All of the NK cells in the decidua expressed high levels of the activation marker CD69 ranging from 42.7% to 94.9%, which is similar to levels observed in αβ and γδ T cells (**Figures 5A, B**, **6C**, **8F**). In contrast, CD69 expression on peripheral blood NK cells never exceeded 39.3% (**Figure 8F**). We also evaluated expression of the sialo-adhesin molecule CD169, a marker of activation, which has been previously reported to be present on >60% of CD16+ decidual NK cells in macaques (48). The frequency of CD169+ NK cells was significantly higher in decidua compared to PBMC, but was only found on 0.4-4.4% of total NK cells in our study (**Figure 8F**).

In a recent single cell RNA sequencing analysis of human first trimester maternal-fetal interface, three NK cells subsets were defined by combinations of NKG2A, NKG2C, CD39, Granzyme B, and CD103 expression (37). These were termed dNK1, dNK2, and dNK3 where dNK1 expressed many HLA-C binding molecules and was the only subset to express LILRB1 that can bind dimeric HLA-G. dNK1 was found to be the subset with most cytotoxic potential by granzyme A, granzyme B, perforin, and granulysin followed by dNK2 and dNK3. We defined dNK1 as NKG2A+, CD39+, granzyme B+ NK; dNK2 as NKG2A+, CD39–, Granzyme B+ NK; and dNK3 as CD103+ granzyme B– NK (**Figure 8G**). Between the 28-color and 18-color flow panels, we could readily identify all three dNK subsets in decidua samples from the rhesus macaques (**Figure 8G**). dNK2 was the most common phenotype in both PBMC and decidua while dNK1 and dNK3 were only consistently found in decidua (**Figure 8G**).

### Myeloid Cells in PBMC and Decidua

**Figure 9:**
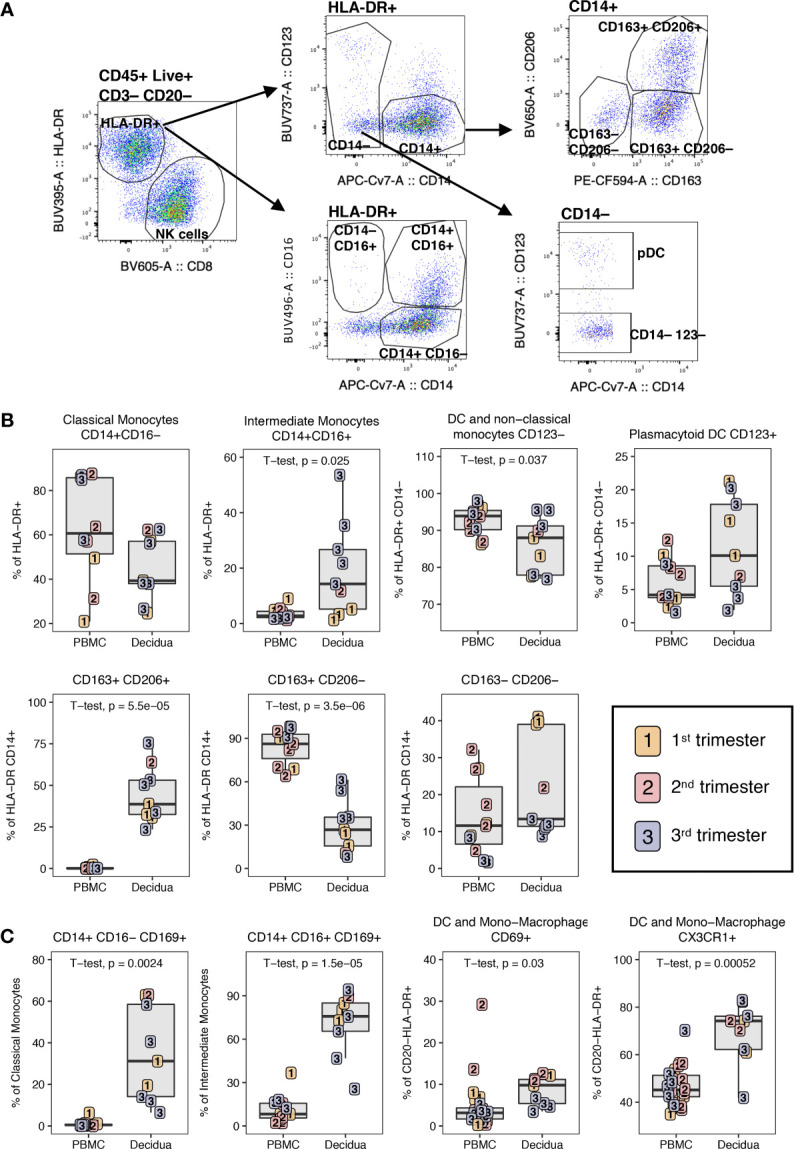
Phenotype of dendritic cells and monocyte/macrophage in PBMC and decidua of normal pregnant rhesus macaques. **(A)** Representative flow plot and gating of HLA-DR+ myeloid cells. **(B)** Frequency of monocyte/macrophage and DC subsets in PBMC and decidua first (yellow), second (red) and third trimester (blue) of normal pregnant rhesus macaques. **(C)** Phenotype of monocyte/macrophages and DCs with box plots showing their frequency. P-values < 0.05 (unpaired t-test) shown on the plots.

Using the gating strategy shown in **Figure 9A** on HLA-DR+ CD3– CD20– CD45+ leukocytes in the 18-color innate panel, we identified a similar proportion of classical monocytes or macrophages (CD14+ CD16–) in PBMC and decidua (**Figure 9B**). CD14+ CD16+ intermediate monocytes were significantly increased in the decidua while non-classical monocytes and dendritic cells (CD14– CD16+/– CD123–) were found at a higher frequency in the PBMC compared to decidua (**Figure 9B**). pDCs, which express CD123, were more abundant in the decidua compared to the PBMC, though not significantly. CD14+ CD163+ CD206+ macrophages were detected at high frequency in the decidua (44.7 ± 16.2%) and were not detected in PBMC (**Figure 9B**). In contrast, single positive CD14+ CD163+ CD206– myeloid cells were predominantly found in the PBMC (84.4 ± 11.3%). Double negative CD14+ CD163– CD206– myeloid cells were highest at the first and second trimester of gestation but were not significantly different between PBMC and decidua (**Figure 9B**). We also assessed the expression of CD169 and CD69 on myeloid cells to determine their activation status. We found that the decidual classical and intermediate monocytes had an increased expression of CD169 (34.2% ± 21.5% and 70.5% ± 20.7 respectively) compared to PBMC (1.0% ± 1.7 and 11.8% ± 9.4 respectively) (**Figure 9C**). DC and monocyte/macrophage subsets (HLA-DR+ CD14– CD123–) in decidua also contained significantly higher frequencies of CD69+ and CX3CR1+ cells when compared to PBMC (**Figure 9C**). These data support increased activation status and migration capability of decidual macrophages and DC populations.

### Changes in Decidual Leukocytes With Gestation Age

To assess temporal gestational effects on decidual immune cell composition, the frequency of major decidual cell populations were correlated with gestational age in days (**Supplemental Figure 1**). Neither T nor B lymphocytes showed a change with gestation (**Supplemental Figure 1**). In contrast, Tregs had a correlative increase with gestation while γδ T cells and iNKTs trended towards an increase (**Supplemental Figure 1**). In the innate compartment, neither total NK cell frequency nor any NK cell subset proportion were significantly different with gestation (**Supplemental Figure 1**). We identified a significant increase in CD14+ CD163+ CD206– decidual macrophages cells with gestation, with a corresponding decrease of CD14+ CD163– CD206– macrophages (**Supplemental Figure 1**). Both pDCs and DC/non-classical monocytes decreased with gestation, though the decrease in pDCs was not significant (**Supplemental Figure 1**).

Among naïve and memory T lymphocyte subsets, several memory CD4+ T lymphocyte subsets showed significant changes with gestational age (**Supplemental Figure 5**). These included gestational dependency of central memory CD4+ T lymphocytes with a highly significant positive correlation (R^2^ 0.8267; p-value >0.001) with increasing gestation age (**Supplemental Figure 5**). This coincided with significant declines in CD4+ Tem, CD4+ TEMRA and activated/proliferating Ki67+ memory CD4+ T lymphocytes in the decidua (**Supplemental Figure 5**). Expression of CCR5 on CD4+ and CD8+ T memory cells also showed significant negative correlation with gestational age (**Supplemental Figure 5**). For Th1-like cells assessed by CXCR3 expression, Th2-like cells assessed by CCR4 expression, and Th17-like cells assessed by CCR6, no correlative relationships were observed with gestational age (**Supplemental Figure 5**). A significant positive correlation between CD4+ memory T lymphocytes PD-1 and HLA-DR expression was observed in the PBMC but not the decidua (**Supplemental Figure 5**) indicating a discordance of activated/exhausted memory CD4+ T between the two compartments.

### PCA Analysis of PBMC and Decidual Leukocytes

**Figure 10:**
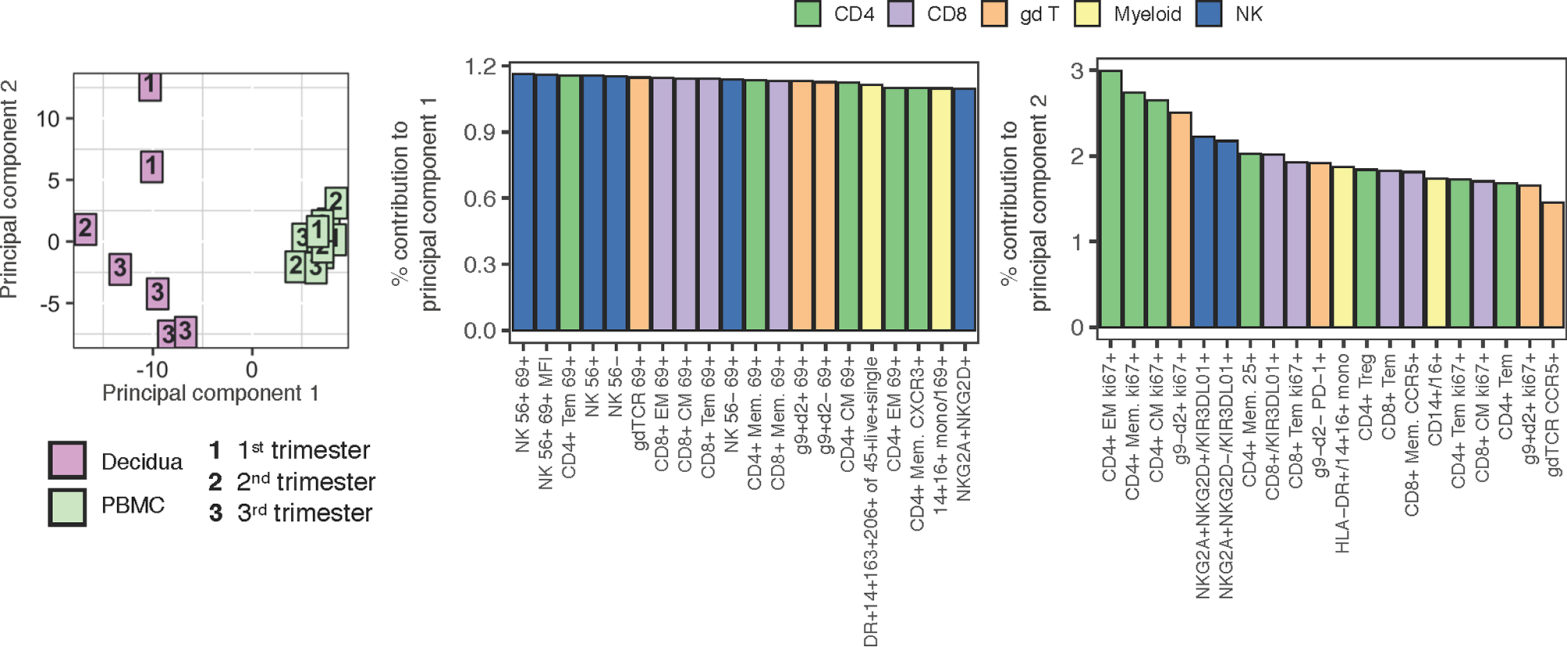
PCA plot using adaptive and innate cell data of PBMC and decidual leukocytes in normal rhesus macaques. A PCA plot was generated using a combination of adaptive and innate immune cells and their features. 217 variables describing frequencies of unique cell types and MFI when applicable (e.g. Granzyme B MFI) were used to contrast PBMC (n = 9) (green) to decidua (n = 7) (pink) samples across 1st, 2nd, and 3rd trimester (left). The top 20 contributing variables to principal component 1 (middle) and principal component 2 (right) are shown and color coded according to major cell populations, CD4+ T (green), CD8+ T (purple), γδ T (orange), myeloid CD20-HLA-DR+ (yellow), and NK cells (blue).

To help interpret what most distinguishes the PBMC and decidual leukocytes in normal rhesus macaques, a Principal Component Analysis (PCA) was performed using all of the populations manually gated from samples stained with both the 28-color adaptive panel and 18-color innate focused panel (**Figure 10**). Despite a range in gestation, the PBMC samples from pregnant macaques clustered tightly in the right-hand quadrant. The decidual leukocytes clustered away from the PBMC by principal component 1 (PC1) and were more spread, indicating their lack of similarity to PBMC and highlighting the heterogeneity of the tissue. Populations which contributed most prominently to PC1 were innate NK cells and many types of T lymphocytes expressing the activation marker CD69. This coincides with differences manifest in the tSNE analysis in **Figures 3**, **7**. By principal component 2 (PC2), the decidual leukocytes are less well-clustered together while a longitudinal pattern of gestational age is apparent. The major contributors to PC2 are memory CD4+ T lymphocyte proliferation markers and frequencies of different innate cells (**Figure 10**).

### ZIKV Impact on Maternal Immunity

**Figure 11:**
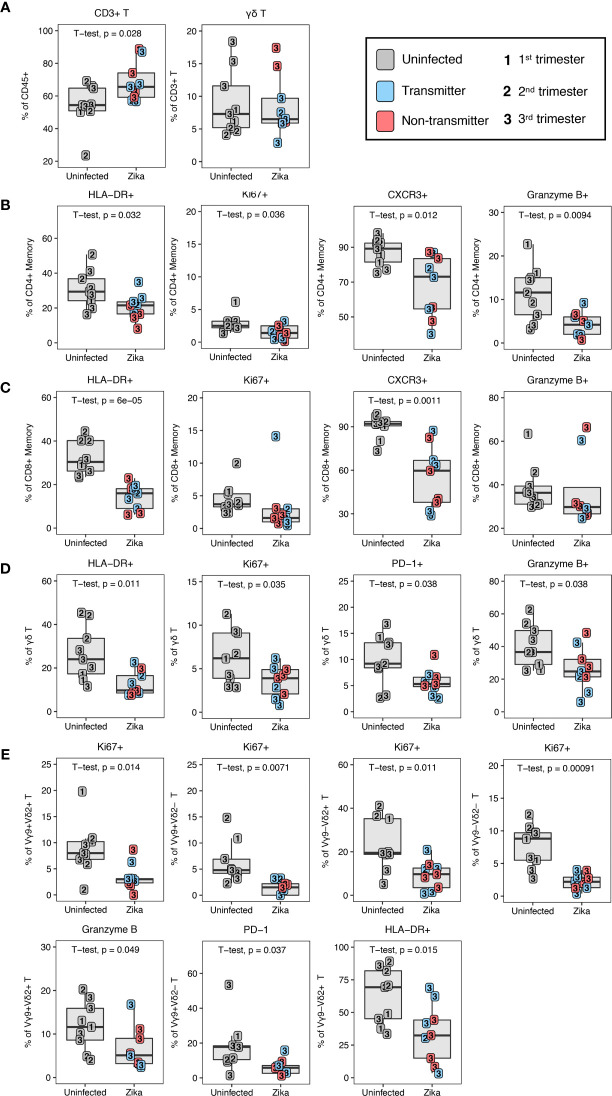
T lymphocyte changes in ZIKV-infected macaques. **(A)** T and γδ T lymphocytes in uninfected (n = 9) (grey) and ZIKV-infected (n = 9) decidual leukocytes. In the ZIKV-infected group, animals with detected amniotic fluid transmission displayed in blue and non-transmitters in red **(B)** Decreased activation of CD4+ T memory in the ZIKV-infected group. **(C)** Decreased activation of CD8+ T memory in the ZIKV-infected group. **(D)** Decreased activation of γδ T lymphocytes in the ZIKV-infected group. **(E)** Decreased decidual γδ T lymphocyte subset activation in ZIKV-infected macaques. P-values < 0.05 (unpaired t-test) shown on the plots.

To investigate the impact of ZIKV on the leukocytes of the peripheral blood and decidua, we studied dams infected with ZIKV during pregnancy in comparison to normal, uninfected rhesus macaques. Placenta was collected from 10 ZIKV-infected dams either at second trimester spontaneous abortion (n=1) or at near term C-section (n=9); details are described in **Supplementary Table 2**. We found that the frequency of total T lymphocytes out of live CD45+ leukocytes was increased in the ZIKV-infected dams compared to the uninfected dams (**Figure 11A**). Within the traditional αβ T cells there were no indications of changes in CD4/CD8 proportions or their memory subtypes to explain the increase in T cells (data not shown).

To determine whether ZIKV infection affected immune function, we assessed the expression of HLA-DR (activation), CXCR3 (activation and trafficking), Ki67 (activation and proliferation), and granzyme B (cytotoxicity) on decidual T lymphocytes in ZIKV-infected dams and compared with decidua of normal pregnancies. A significant decline in HLA-DR+ activated memory CD4+ and CD8+ T lymphocytes was observed in ZIKV-infected decidua as compared to normal decidua (**Figures 11B, C**). Coinciding with the lack of HLA-DR, Ki67+ memory CD4+ T lymphocytes were also significantly decreased in the infected animals compared to normal decidua (**Figure 11B**). Other notable changes on memory T lymphocytes in decidua of ZIKV-infected dams including a significant decrease in frequency of CXCR3 positive cells in both CD4+ and CD8+ T lymphocytes, and reduced frequency of granzyme B-positive CD4+ T memory cells compared to uninfected decidua (**Figures 11B, C**). A similar pattern of perturbation was observed in decidual γδ T cells of ZIKV-infected dams, namely a significant decrease in HLA-DR, Ki67, PD-1, and granzyme B, indicative of inhibition of activation, proliferation and cytotoxicity of γδ T (**Figure 11D**). Vγ9 and Vδ2 subsets likewise showed a general reduction of proliferative and activation potential by Ki67+ suggesting that the effect was global and not confined to a particular subset of γδ T (**Figure 11E**). In contrast to Ki67, decreased granzyme B expression was confined to the Vγ9+ Vδ2+ subset of γδ T (**Figure 11E**). This subset was recently shown to have the greatest cytotoxic potential and cytokine secretory capacity in healthy adults (49). In addition, decreased PD-1 expression and HLA-DR was confined to the Vγ9+ Vδ2– and Vγ9– Vδ2+ subsets respectively.

**Figure 12:**
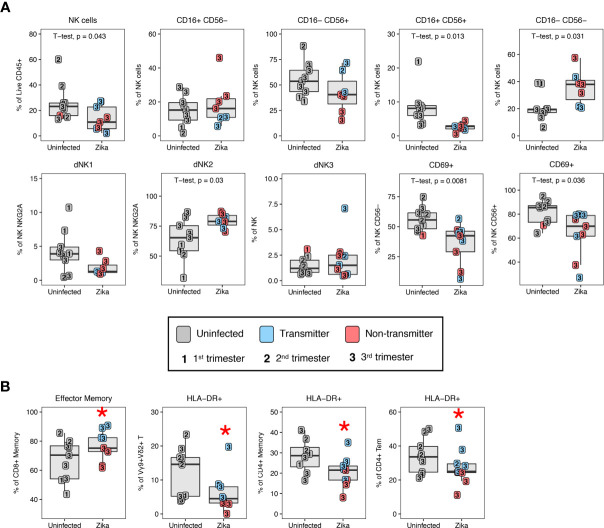
Changes in decidual NK cells in ZIKV-infected dams and immune correlates of transmission. **(A)** Altered NK cell subsets by CD16/CD56, granzyme B, KIR3DL01, and CD169 in the decidual leukocytes of uninfected (n=9) (grey) and ZIKV-infected (n=9) dams. In the ZIKV-infected group, animals with detected amniotic fluid transmission displayed in blue and non-transmitters in red. P-values < 0.05 (unpaired t-test) shown on the plots. **(B)** Immune parameters significantly different between transmitter and non-transmitter dams. P-value <0.05 between transmitters and non-transmitters shown as red asterisk. Normal decidual leukocytes are displayed for comparison.

To study changes in the innate compartment, we found a loss of total NK cells in ZIKV-infected compared to uninfected dams (**Figure 12A**). These decreases were subset specific, in that the decidual CD16+ CD56+ NK subset showed a significant decrease whereas the CD16– CD56– NK cell subset showed a proportional increase in frequency in ZIKV-infected compared to uninfected macaques (**Figure 12A**). We observed an increase in dNK2 in ZIKV-infected dams while dNK1 and dNK3 were comparable to normal decidua (**Figure 12**). Similar to the changes observed in T cells of ZIKV-infected macaques, CD69 expression on both CD56– and CD56+ NK cells were reduced compared to normal decidua (**Figure 12A**). We did not observe increased CD169 expression on NK cells contrary to a recent study that reported an increase in CD169-positive CD16+ NK cells in the decidua of ZIKV-infected dams (48). Few changes in myeloid cell proportions were observed following ZIKV-infection, although CD14+ CD163– CD206– cells were higher in the decidua of uninfected macaques compared to ZIKV-infected macaques (22.0 ± 13.1% and 8.5 ± 6.4%, respectively, p = 0.025).

Similar to changes observed in the decidua, the majority of changes observed in PBMC of ZIKV-infected dams were confined to the T lymphocyte compartment and manifest as a decrease in markers of activation of HLA-DR, Ki67, and PD-1 expression on CD4+ and CD8+ T lymphocyte subsets compared to normal pregnant macaques (**Supplemental Table 4**). In addition to the reduced activation of αβ T lymphocytes, γδ T lymphocytes appeared to be more differentiated by increased TEMRA memory phenotype and the Vγ9+ Vδ2+ had a reduced activation status. Both the CD56– NK cells, and myeloid cells (HLA-DR+ CD20–) showed decreased Ki67 compared to uninfected macaques (**Supplemental Table 3**).

### Global Analysis of the Impact of ZIKV on the Maternal-Fetal Interface

**Figure 13:**
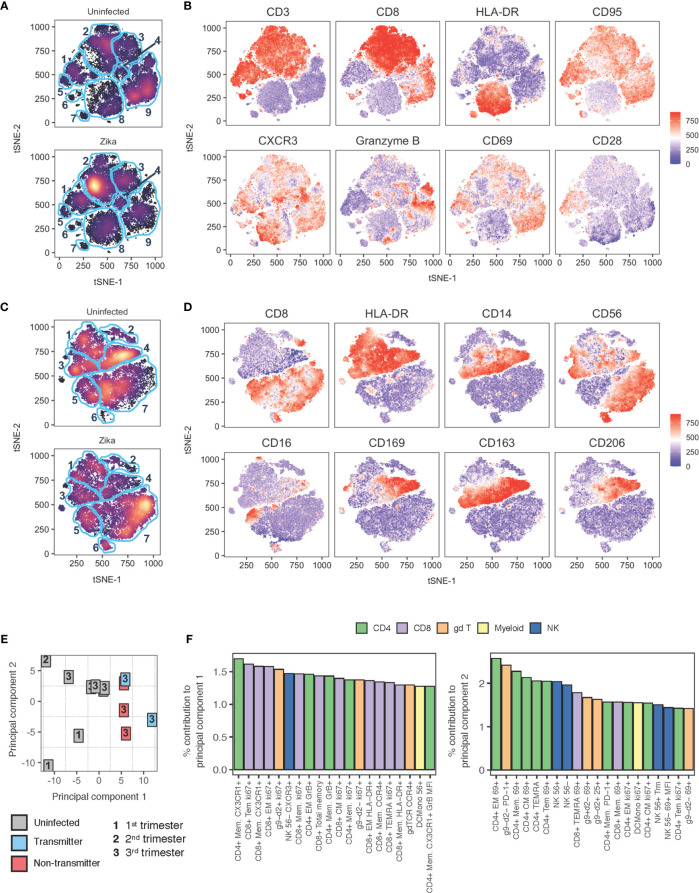
tSNE and PCA analysis of decidual leukocytes of uninfected and ZIKV-infected rhesus macaques. **(A)** tSNE plot representing an equal number of CD45+/live/single cells/leukocytes cells from decidua of rhesus macaques experiencing a normal pregnancy (n = 9) or after ZIKV infection (n = 8). The 28-color adaptive panel was used to generate this plot. Blue gates were manually drawn based on clustering patterns. **(B)** Individual MFI gradients of eight markers on the tSNE map. Red coloring represents high MFI and blue coloring represents low MFI. **(C)** tSNE plot representing an equal number of CD45+/live/single cells/CD3-/CD20- cells from decidua of rhesus macaques experiencing a normal pregnancy (n = 8) or after ZIKV infection (n = 7). The 18-color innate panel was used to generate this plot. Blue gates were manually drawn based on clustering patterns. **(D)** Individual MFI gradients of eight markers on the tSNE map. Red coloring represents high MFI and blue coloring represents low MFI. **(E)** PCA plot of decidual leukocytes of uninfected and ZIKV-infected dams. **(F)** The top 20 contributing variables to principal component 1 (left) and principal component 2 (right) are shown and color coded according to major cell populations, CD4+ T (green), CD8+ T (purple), γδ T (orange), myeloid CD20-HLA-DR+ (yellow), and NK cells (blue).

To further understand the impact of ZIKV on pregnancy and specifically in the decidua, we performed tSNE and PCA analysis of the decidual leukocytes in comparison to uninfected animals (**Figures 13A–C**). A tSNE analysis of decidual cells computed with data from a 28-color panel focused on the adaptive immune system resolved several notable differences (**Figures 13A, B**). In particular, clusters 2 and 8 appear to be affected by ZIKV infection (**Figure 13A**). Cluster 2 is consistent with CD8 memory T cells by CD3+ CD8+ CD95+ CD28– cells, and cluster 8 are likely myeloid cells defined by CD3– HLA-DR+ CD20– expression (**Figure 13B** and **Supplemental Figure 6**). An additional 16 markers used to compute the tSNE are found in **Supplemental Figure 6**.

tSNE analysis of innate markers using an 18-color panel focusing on the innate immune system further highlighted differences in decidual immune composition after Zika virus infection (**Figures 13C, D**). Prior to computing the tSNE map, dead cells and CD45+ CD3+ or CD45+ CD20+ cells were excluded. The most notable differences appear in clusters 4, 6, and 7. Cluster 4 are tissue macrophages defined by their HLA-DR+ CD14+ CD163+ CD206+ CD39+ CD169+ phenotype (**Figures 13C, D** and **Supplemental Figure 7**). Cluster 7 are CD16– CD56+ NK cells defined by the presence of CD8+ HLA-DR–/lo CD16– and CD56+, and cluster 6 are also NK CD16– CD56+ NK cells with the additional expression of CD39 (**Figures 13C, D** and **Supplemental Figure 7**).

PCA analysis shown in **Figure 13E** determined immune cell subsets that most contributed to the separation between infected and uninfected decidua. The uninfected decidua is found to cluster away from infected tissues by PC1. The top contributors to PC1 were memory T lymphocytes expressing activation and proliferation markers (**Figure 13F**). PC2 did not well separate uninfected from infected samples (**Figure 13E**). Little to no clustering effect was observed between animals with congenital transmission and no congenital transmission of ZIKV (**Figure 13E**). Like the uninfected decidua, there was also wide heterogeneity among ZIKV-infected decidua.

### ZIKV Non-Transmitters Compared to Transmitters

Five of the 10 ZIKV-infected dams had ZIKV RNA detected by PCR in the amniotic fluid. Although we cannot definitively exclude absence of placental transmission in the PCR-negative dams, we compared immune parameters in the amniotic fluid ZIKV PCR positive (transmitters) and negative (non-transmitters) dams. The frequency of single positive CD16– CD56+ NK cells in the non-transmitter dams (24.4 ± 14.1%) was reduced compared to the transmitter dams (59.7 ± 12.2% (**Figure 12A**). Animals that transmitted also had higher frequencies of CD8+ T effector memory cells compared to non-transmitters (83.5 ± 6.3% compared to 68.2 ± 5.9%, respectively) (**Figure 12B**). Interestingly, macaques that did not transmit ZIKV had the greatest reduction of T cell activation compared to both uninfected and ZIKV-transmitters (**Figure 12B**). For example, HLA-DR expression in Vγ9+ Vδ2+ was reduced from 9.2 ± 5.6% to 2.4 ± 1.4% in non-transmitters, and HLA-DR expression on total CD4+ memory T cells was reduced from 24.6 ± 6.0% to 15.2 ± 4.8% in non-transmitters (**Figure 12B**). CD4+ Tem cells also had a reduced expression of HLA-DR in non-transmitters compared to transmitters (**Figure 12B**).

## Discussion

This is the first comprehensive single-cell phenotypic analysis of multiple immune cell subsets in the decidua and peripheral blood of healthy rhesus macaques across the three trimesters of pregnancy. By simultaneously probing conventional T lymphocytes, iNKT, Tregs, γδ T, B lymphocytes, NK cells, monocytes, macrophages and dendritic cells (DC), we could evaluate components of both the innate and adaptive immune system and study their interaction in the setting of normal pregnancy and after ZIKV infection of pregnant macaque. Several findings reported in blood and decidua of human pregnancy were recapitulated in normal rhesus macaque pregnancy reinforcing the value of this NHP model for the study of congenital viral infections. Notable among the similarities between human and NHP decidua were the predominance of the CD16–CD56+ phenotype of NK cells, enrichment of CD4+ Tregs, enrichment of memory CD4+ and CD8+ T lymphocytes, increased activation but decreased cytotoxicity of decidual NK cells and CD8+ T lymphocytes, a predominance of CD206+ macrophages, and a paucity of B lymphocytes (6, 20, 50–52). tSNE analysis of CD45+ live leukocytes on the 28-color adaptive lymphocyte-focused flow cytometry panel and on CD3–CD20–CD45+ live leukocytes on the 18-color innate cell-focused flow panel revealed several clusters representing populations of CD4+ and CD8+ T lymphocytes, NK cells, monocytes or macrophages and DCs that were unique to either peripheral blood or decidua. The immune cell diversity between decidua and PBMC was similar to what was recently reported in humans using a similar approach (53).

Memory T lymphocytes comprise roughly 5-20% of the CD45+ leukocyte population in human decidua from the first trimester and increase with gestational age. As part of the adaptive immune system they are key players in mounting an immune response against foreign antigens in pregnancy (10, 22). Decidual CD8+ T lymphocytes are unique in that they recognize fetal antigens but remain tolerant to avoid fetal rejection. However, their role in pathogen-specific immunity and the properties of the immune response necessary to clear infections without harming the fetus are not known (23). In our study, we performed an extensive evaluation of the memory phenotype, activation status, cytotoxic granule content, and chemokine receptor expression on memory subsets of both circulating and decidual CD4+ and CD8+ T lymphocytes in pregnancy. T lymphocytes were the dominant leukocyte population in the decidua across all three trimesters with the exception of one second trimester dam in whom NK cells comprised roughly 60% of the decidual leukocyte population (**Figure 2B**). Reciprocally, we did not find NK cells as the dominant decidual population in first trimester. Neither was there a clear trend of decidual NK cells declining with gestation age. These findings are in contrast with humans where NK cells are the dominant leukocyte population in first trimester decidua. Whether this observation represents a species-specific difference in decidual leukocyte composition will require further study in a larger cohort of animals.

Relative to studies on decidual NK cells, data on decidual T lymphocytes are limited particularly for rhesus macaque decidua. A recent study in rhesus macaques at the third trimester of pregnancy used mass cytometry to examine multiple immune subsets including memory T lymphocytes in choriodecidual cells in decidua parietalis as well as in placental villi (54). The decidual leukocytes evaluated in our study were primarily isolated from tissue removed from the maternal surface of the placenta (decidua basalis) and stripped away from chorionic tissue. Using a combination of CD95, CD28, CCR5, CD45RA, CD69 and CD103, we delineated CM, EM, TEM, TEMRA, and tissue-resident Trm memory. Rhesus macaques had significantly higher proportions of EM CD8+ and CD4+ T in the decidua compared to peripheral blood consistent with human data (20, 25, 55). Less clear is the concordance between rhesus macaque and human decidua for other memory subsets. CM CD8+ and CD4+ T lymphocytes were present at significantly lower frequencies in rhesus macaque decidua. One study showed higher frequencies of CM CD4+ and CD8+ T lymphocytes in term human decidua compared to peripheral blood (55). CD4+ TEMRA lymphocytes did not differ between peripheral blood and decidua; however, CD8+ TEMRA were significantly lower in the decidual compartment, contrary to human studies showing a higher proportion of CD8+ TEMRA in the decidua (22). It is noteworthy that in rhesus macaques, the proportion of decidual CM CD4+ T was lowest in the first trimester and highest in the third trimester, a finding that has not previously been reported. The increase in decidual CM CD4+ T with increasing gestation age was associated with a decline in Ki67+ activated memory CD4+ T (both CM and EM subsets) and a decline in CCR5+ CD4 memory and CD8 memory. These changes with gestational age were confined to the decidua and not observed in circulating lymphocytes. Reduced decidual CCR5+ memory CD4+ T with gestation age may have implications for the relative risk of mother-to-child transmission of HIV in different trimesters of pregnancy (56).

Consistent with findings in humans, decidual EM, CM and TEMRA CD8+ T had lower granzyme B content compared to their circulating memory counterparts (20). Of note, the proportion of granzyme B+ TEM and CM CD8+ T lymphocytes was significantly higher in the decidua but their granzyme B content measured by MFI was lower. Thus, decidual CD8+ T contain substantial proportions of Granzyme B-positive cells but likely are less cytotoxic compared to circulating memory CD8+ T lymphocytes. With respect to effector function, the majority of decidual memory CD4+ and CD8+ T were highly activated and skewed to a Th1 phenotype as evidenced by high proportions expressing CD69, HLA-DR, and CXCR3. This was in striking contrast to circulating memory T lymphocytes that were less activated and showed a mixture of Th1 (CXCR3+), Th2 (CCR4+), and Th17 (CCR6+, CCR4+) cells and is similar to findings in humans (55). In concert with high levels of activation, decidual CD4+ and CD8+ T lymphocytes contained significantly higher levels of PD-1+ cells compared to circulating memory T lymphocytes but not in a correlative manner like the PBMC. This is not surprising in light of studies showing expression of immune inhibitory check-point receptors on human decidual T lymphocytes as one likely mechanism by which decidual T lymphocytes maintain concurrent effector function and a tolerogenic phenotype during pregnancy (21, 57).

In addition to conventional T lymphocytes, CD4+ Tregs, iNKT and γδ T lymphocytes accounted for roughly 20% of the total decidual T (CD3+) lymphocyte population. Tregs play an essential immunosuppressive role in inhibiting alloreactive T cells and maintaining tolerance during normal pregnancy. As in humans, CD4+ CD25bright CD127lo Tregs in rhesus macaques were present at significantly higher frequencies in the decidua compared to peripheral blood and showed a significant increase with gestation age. Human studies of normal pregnancy have shown that decidual Treg frequencies remain unchanged (d. basalis) or increase (d. parietalis) at term gestation (51, 58–60). A recent study provided evidence for clonally expanded effector Tregs, likely containing fetal antigen-specific Tregs, increasing in the 3^rd^ trimester of normal pregnancy but decreasing in preeclampsia (61). While we did not characterize the type of decidual Tregs in this study, the findings of CD4+ Tregs enrichment in macaque decidua and its relationship to gestation age appear similar to human decidua.

Among other immunomodulatory T lymphocytes, low frequencies of decidual iNKT were detected at levels comparable to peripheral blood. It should be noted that the method of iNKT detection is an important consideration for comparison with studies reporting on NKT lymphocytes. Several publications including one on macaque decidua have defined NKT as CD3+CD56+ T lymphocytes (62). This is erroneous and does not represent genuine iNKT lymphocytes (63). We used T lymphocyte expression of the Vα24 TCR along with binding to α-galactoslyceramide(GC)-loaded CD1d tetramers for specific and stringent identification of iNKT lymphocytes as described in humans and nonhuman primates (43, 64). In our study, CD3+CD56+ T lymphocytes were enriched in the decidua with mean frequency of 21.7% of T lymphocytes as compared to <5% of T lymphocytes in peripheral blood. Yet, less than 1% of decidual CD56+ T were genuine iNKT as defined by Vα24+ T lymphocytes binding to αGC-loaded CD1d tetramer. Moreover, all Vα24+ CD1d-tetramer-positive iNKT lymphocytes did not express CD56. In two studies in humans that used stringent criteria for identification, iNKT were detected in first trimester decidua at low frequencies of <0.5% of CD3+ decidual leukocytes and were present at significantly higher frequencies in the decidua compared to peripheral blood (41, 42).

γδ T lymphocytes are another unconventional T lymphocyte subset that recognize non-peptide antigens in an MHC-independent manner and respond to bacterial and viral infections (65). Early trimester decidua is enriched for γδ T but their role in pregnancy is not well understood (46). In utero CMV infection is associated with expansion of fetal Vγ9– γδ T lymphocytes (66). In one study ZIKV infection in humans was associated with expansion of Vδ2+ γδ T in the peripheral blood (67). To our knowledge there are no published data on decidual γδ T in rhesus macaques. Using a NHP cross-reactive pan-γδ TCR antibody along with antibodies specific for the Vγ9 and Vδ2 TCR chain, four populations of γδ T were defined of which the Vγ9–Vδ2– subset formed the bulk of the γδ T lymphocytes in the decidua and peripheral blood. γδ T lymphocytes showed higher frequencies in the decidua particularly at third trimester gestation. Of total γδ T cells, the Vδ2 subset is significantly higher in blood of healthy human donors compared to early gestation decidua (46). The same conclusion can be inferred in rhesus macaques as the dominant Vγ9+Vδ2+ population was significantly higher in the peripheral blood compared to the decidua. Similar to the conventional T lymphocytes, decidual γδ T were highly activated and enriched for effector memory cells in the decidua. These data suggest that despite being a relatively small population, decidual γδ T also have the capability to be potent effectors. Their role in protective immunity at the maternal-fetal interface remains to be determined. Of interest, ZIKV-infected pregnant dams in our study had increased levels of circulating Vδ2+ γδ T compared to normal pregnancies. In the ZIKV-infected dams, the decidual γδ T had a reduced proliferation potential similar to the memory CD4+ T but also reduced PD-1 expression. This may suggest that decidual γδ T are less exhausted and could have effector function in the face of a viral infection.

Among innate leukocytes we evaluated NK cells, monocytes/macrophages and dendritic cells in the decidua and peripheral blood of normal pregnancy. Based on co-expression of the scavenger receptor CD163 and the mannose receptor CD206, three populations of decidual macrophages were detected of which the dual positive CD163+ CD206+ subset was only seen in the decidua. A CD163+ CD206– decidual macrophage population was present but at a significantly lower frequency compared to peripheral blood where this monocyte subset was dominant. The two populations of CD163+ decidual macrophages appear to be phenotypically analogous to the CD11c(lo) and CD11c(hi) subsets with distinct gene expression profiles described in humans (50). Although neither subset is precisely M1- or M2-polarized, the gene profile of the CD11c(lo) CD206+ subset resembles M2-polarized macrophages. It is noteworthy that the CD163+CD206– decidual macrophage population increased with gestational age as did the frequency of CD14+CD16+ inflammatory monocytes. These changes likely reflect the plasticity of the decidual macrophages moving to a more inflammatory phenotype as pregnancy progresses to term.

NK cells are the most abundant decidual leukocyte population in the first trimester of pregnancy and essential for implantation of the placenta through their interactions with extravillous trophoblasts. Decidual NK cells are phenotypically and functionally distinct from NK cells in the circulation and at other tissue sites and constitute a diverse population of tissue-resident innate lymphoid cells (68). Recent studies on first trimester human decidua using single cell RNAseq and mass cytometry have revealed several novel populations including dNK1, dNK2, and dNK3 based on their gene expression profile, phenotypic markers and function (11, 37). In this study we complemented evaluation of macaque NK subsets based on CD16 and CD56 co-expression patterns with NKG2A, CD39, CD103 and Granzyme B to define putative dNK1, dNK2 and dNK3 populations in macaques. These definitions were adapted from and based on the single cell gene expression, flow cytometry and mass cytometry profile of human first trimester decidua (11, 37). By flow cytometry, we defined dNK1 as NKG2A+ CD39+ granzymeB+ NK; dNK2 as NKG2A+ CD39+ granzymeB+ NK; and dNK3 as CD103+ granzymeB– NK. CD39 is a surface bound ectonucleosidase enzyme which is upregulated during inflammatory conditions. It is constitutively expressed in the placenta and is found on many immune cell subsets such as Tregs, NK cells, monocyte/macrophages, and dendritic cells. CD39 along with CD73 can convert ATP to adenosine leading the extracellular environment from a proinflammatory to immunosuppressive environment (69). tSNE analysis of the 28-color and 18-color flow cytometry panel revealed several unique non-overlapping clusters for NK cells in decidual and circulating leukocytes. With manual gating, several expected and novel observations were made. Consistent with previous studies in humans and macaques, the majority of decidual NK cells were CD56+CD16– (7, 9, 15, 16). This was in contrast to circulating NK cells in macaques that are predominantly CD16+CD56– (38). Macaque decidua also contained a clearly discernible population of CD16+ CD56+ double-positive NK cells that were not detected in peripheral blood. A similar double-positive NK population has been reported in vaginal and rectal mucosal tissues of rhesus macaques (70). To our knowledge this is the first report of CD16+CD56+ NK cells in macaque decidua. Similar to humans, granzyme B content in the decidual NK subsets was lower than their circulating phenotypic counterparts. When analyzed for dNK1-3 subsets on the basis of CD39, granzyme B and CD103 expression, the dominant dNK2 population was significantly lower in the decidua compared to peripheral blood. The dNK1 and dNK3 subsets constituted a small population of NK cells which was present at higher frequencies in the decidua. The dNK1 and dNK3 decidual populations were lower in the third trimester compared to the first trimester, whereas the dNK2 decidual population increased from the first to the third trimester. These data are consistent with the known functions of human dNK1 cells expressing high levels of KIRs and interacting with HLA-C molecules on extra-villous trophoblasts to promote placentation in the first trimester (11). Macaque decidual NK cells also contained a high frequency of NKG2A+NKG2D+ NK cells that were absent or detected at low frequencies in the circulation. NKG2D is a C-type lectin-like activating receptor expressed on NK cells and T lymphocyte subsets. In a study of first trimester human decidua, NKG2D was expressed on all CD56+ decidual NK and surface expression of the NKG2D ligands, UL16 binding protein and the stress-inducible MHC-Class I related chain molecules (MIC), was detected on extra-villous trophoblasts, villous trophoblasts, and decidual macrophages (71). A significant increase in NKG2D expression was observed in second trimester decidual NK as compared to first trimester (72). NKG2D was also shown to be involved in the cytotoxic effector function that decidual NK acquire *in vitro* on exposure to human CMV-infected autologous fibroblasts (73). Because the anti-NKG2A antibody in rhesus macaques does not differentiate between the inhibitory receptor NKG2A and the activating receptor NKG2C, we cannot be certain whether the NKG2D+ decidual NK cells co-express NKG2A or NKG2C (74). However, in light of the human data, it is likely that the NKG2A+NKG2D+ NK population in macaque decidua is a cytotoxic population. Similar to the T lymphocyte subsets in macaque decidua, the majority of decidual NK cells were significantly more activated than circulating NK cells as evidenced by surface expression of CD69, CXCR3 and CD169 molecules. The chemokine receptor CX3CR1 was also expressed on decidual NK cells but at lower frequencies compared to circulating NK cells.

Our analysis of decidual and circulating leukocytes from a ZIKV infection study in pregnancy in comparison to normal decidua yielded unexpected and interesting findings. In human studies, patients with ZIKV infection are reported to have increased DN T cells and increased activated CD8+ T cells when compared to healthy controls. Furthermore, nonclassical T cell subsets such as double negative (CD4-CD8-) Vδ2 TCR+ T cells are increased in ZIKV infected individuals which correlate with acute resolution of symptoms. This suggests that Vδ2 TCR+ T cells play a role in resolution in ZIKV symptoms (67). Based on these data, ZIKV could potentially disturb the balance at the maternal-fetal interface, as early gestation decidua has an increased frequency of γδ T cells compared to matched maternal blood (46). *In vitro* studies have shown that ZIKV can infect and replicate in human placental macrophages or Hofbauer cells, viral replication coincides with type I interferon induction, and anti-viral gene expression and pro-inflammatory cytokines (75). The mechanism of transmission is thought to by direct infection of Hofbauer cells and disrupting placental barrier. The role of decidual immunity is unknown as few studies have investigated tissue level cellular immunity to ZIKV despite its broad tissue trophism (76). To our surprise, the most striking finding in decidual cells from ZIKV-infected dams was a decrease in NK cells and increase in total T lymphocytes with significant reduction in activated (HLA-DR+ and/or Ki67+) memory CD4+ and CD8+ T and γδ T lymphocytes, reduced frequency of decidual Granzyme B+ CD4+ T and γδ T lymphocytes and reduction in CXCR3+ memory CD4+ and CD8+ T lymphocytes. We also saw a reduction in circulating DN T lymphocytes and a significant reduction in activated, proliferating, and PD-1-positive memory CD4+ T, memory CD8+ T, γδ T, and NK cells in ZIKV-infected dams as compared to healthy pregnant macaques. Overall, these data suggest an immunosuppressive effect with suppression of inflammation and decreased immune recruitment of decidual memory T cells at the maternal-fetal interface. Our findings raise the possibility that the prolonged ZIKV viremia reported in pregnant humans and macaques (29, 77, 78) is related to the immunosuppressive effect of ZIKV infection. Immunosuppression has been reported with Asian-lineage ZIKV related to suppression of type I interferon responses and induction of a M2 anti-inflammatory type transcription phenotype of monocytes ex vivo. But no changes in T cell phenotype were observed in this study (79).

The immunosuppressive effects we have observed in this study are in contrast to the autoimmunity (loss of immune suppression) type of symptoms by Guillain-Barré syndrome that may arise in conjunction with ZIKV infection (76). It is possible that ZIKV induces autoimmunity of the circulating leukocytes while at the tissue-level of the already tolerant maternal-fetal interface, the immune cell environment is silenced by loss of T cell activation and reduced infiltration of CXCR3-expressing CD4+ and CD8+ T lymphocytes which are inflammation-homing T cells. The CXCL10-CXCR3 axis is important in attracting effector T cells to decidual tissue (23). In mice epigenetic silencing of T cell chemoattractant genes in decidual stromal cells led to impaired accumulation of decidual effector T lymphocytes (80). CXCR3 blockade eliminated decidual CD8+ T cell infiltration and protected against in utero fetal infection and immunopathogenesis in a murine model of Listeria infection (81). The question of whether or not the immunosuppressive effects in the maternal decidua reflected an impaired virus-specific cellular immune response or protected against immunopathology are unresolved and will require future prospective studies.

The overall picture of the NHP maternal-fetal interface emerging from our study is that of a complex, dynamic immune environment with capability of robust effector activity balanced with features of a tolerogenic phenotype. Normal macaque decidua is populated with a dominance of memory T lymphocytes, CD8+ more than CD4+, throughout the gestation period with a subset showing markers of tissue-resident memory. The majority of memory CD4+ and CD8+ T, and γδ T lymphocytes are highly activated with a CXCR3+ Th1 phenotype but a significant proportion also express PD-1. Higher frequencies of cytotoxic memory T lymphocytes with less granzyme B content at the single cell level compared to their circulating counterparts are detected in the decidua. Decidual NK cells are phenotypically distinct with a dominance of cytotoxic CD56+ NK cells that co-express NKG2D and NKG2a/c. Similar to memory T lymphocytes, the majority of decidual NK cells are activated and express CXCR3 but have lower granzyme B content compared to their circulating counterparts. In the myeloid lineage, we found a population of tissue-resident CD163+ CD206+ macrophages which were unique to the decidual environment. We also found a strong presence of tolerogenic CD4+ Tregs which correlated with gestational age in the decidua. In all, these features identify the maternal-fetal interface as a distinct and unique environment that pathogens need to navigate for vertical transmission to the fetus. Our findings of reduced activation, reduced CXCR3 expression and reduced cytotoxicity of decidual memory T lymphocytes in ZIKV infection indicate local immunosuppression and impaired immune recruitment as possible mechanisms of vertical transmission.

In conclusion, we have reported on the first deep and comprehensive analysis of immune cells at the maternal-fetal interface and its comparison to circulating leukocytes in a normal rhesus macaque pregnancy model. By extending our analysis to an investigation of changes in the maternal decidua in a cohort of ZIKV-infected dams, we provide novel insights in to immune perturbations following a congenital viral infection. The stark contrasts of the circulating to decidual leukocytes highlights the immunological barrier formed by the maternal-fetal interface and the need to study this in the context of pathogenic infections that can be transmitted from mother to fetus. Such studies are needed to find vaccine targets against congenital infections.

## Data Availability Statement

The raw data supporting the conclusions of this article will be made available by the authors, without undue reservation.

## Ethics Statement

The animal study was reviewed and approved by TNPRC and ONPRC IACUC.

## Author Contributions

MM and AK conceived the study. MM, MF, and AK wrote the manuscript. MM, AK, MF, JH, VR, and NM reviewed the manuscript. MM, LS, ES, DT, DS, JH, and VR helped procure and process samples. MM, LS, and ES performed the experiment. MM, MF, and AK analyzed the data. MM and MF prepared and processed the data for presentation. AK, JH, VR, and NM were responsible for the support of experiments. All authors contributed to the article and approved the submitted version.

## Funding

Funding was provided by NIH by 1P01AI129859, TNPRC Base Grant OD011104, and ONPRC Base Grant OD011092. Additional funding for the ZIKV-infected study group was provided by the Bill and Melinda Gates Foundation (OPP1152818). Animal samples were available through the ONPRC Pathology tissue distribution program, which is supported by ONPRC NIH Base Grant OD011092.

## Conflict of Interest

The authors declare that the research was conducted in the absence of any commercial or financial relationships that could be construed as a potential conflict of interest.

## Publisher’s Note

All claims expressed in this article are solely those of the authors and do not necessarily represent those of their affiliated organizations, or those of the publisher, the editors and the reviewers. Any product that may be evaluated in this article, or claim that may be made by its manufacturer, is not guaranteed or endorsed by the publisher.
