# Supplementary material for: Immune Profile of the Normal Maternal-Fetal Interface in Rhesus Macaques and Its Alteration Following Zika Virus Infection
Source: Front Immunol. 2021 Jul 29;12:719810. doi: 10.3389/fimmu.2021.719810 (PMC8358803; doi:10.3389/fimmu.2021.719810)
Supplement: Supplementary file 1 [file Presentation_1.pptx]

## Slide 1
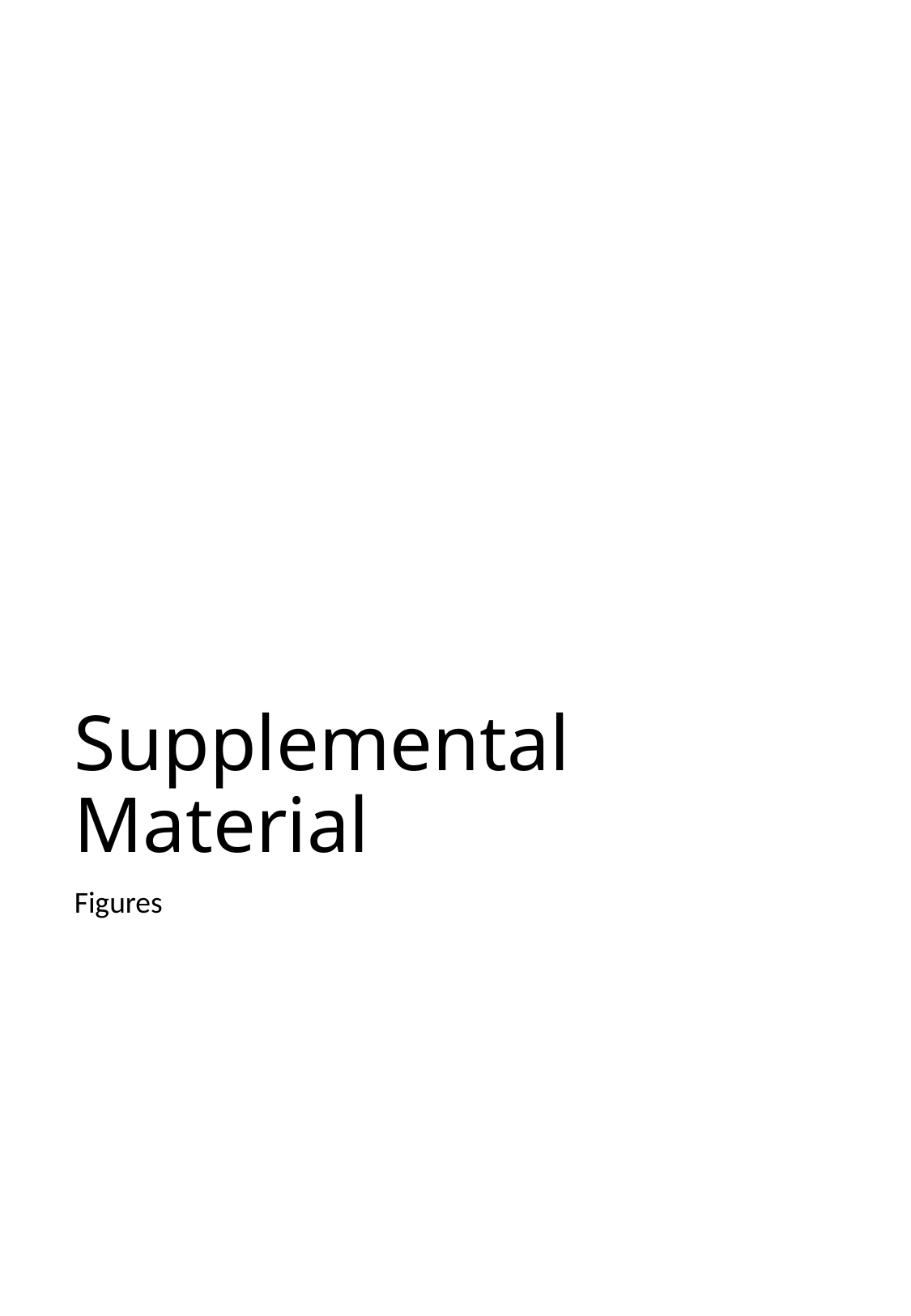

# Supplemental Material
Figures

## Slide 2
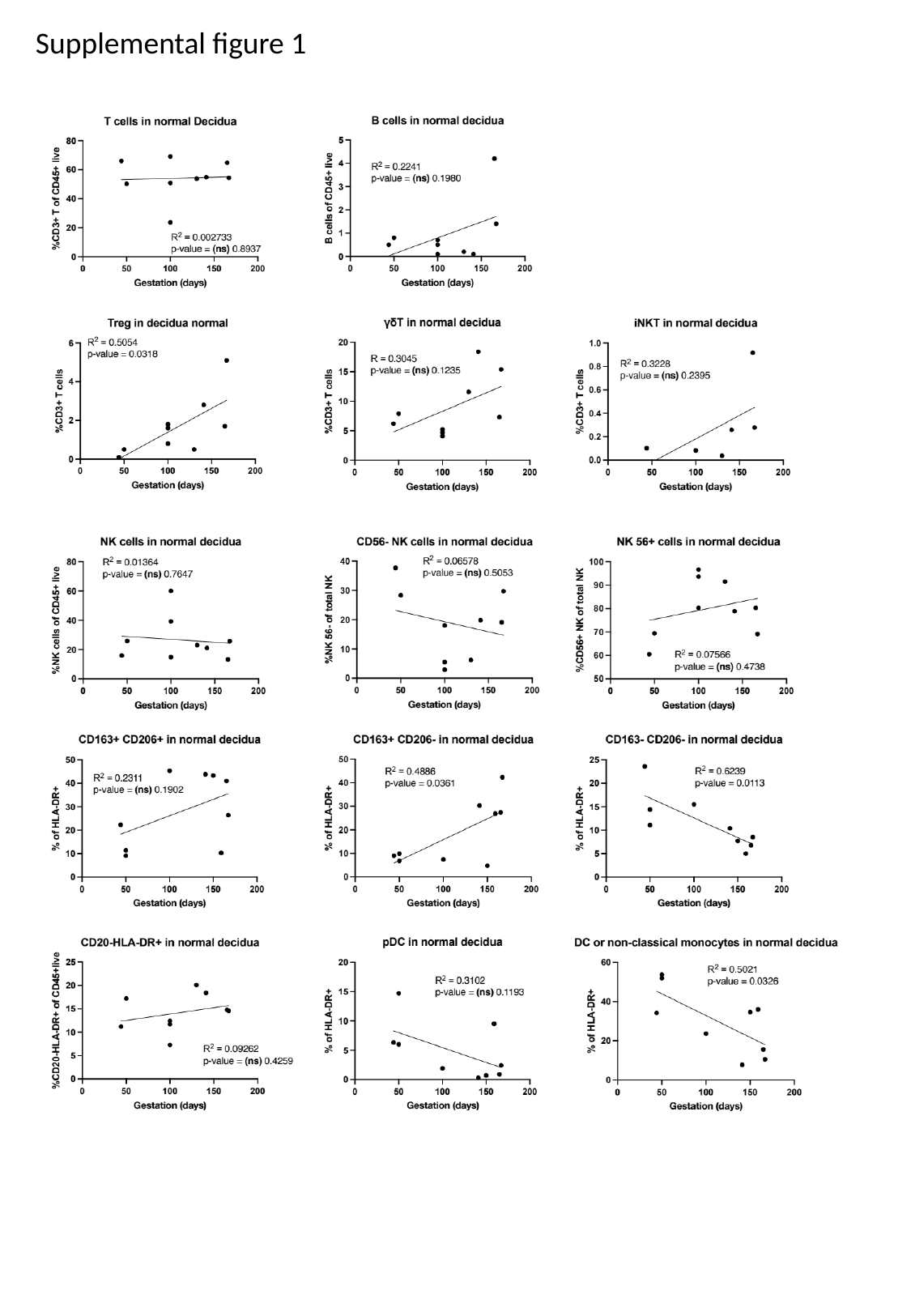

Supplemental figure 1

## Slide 3
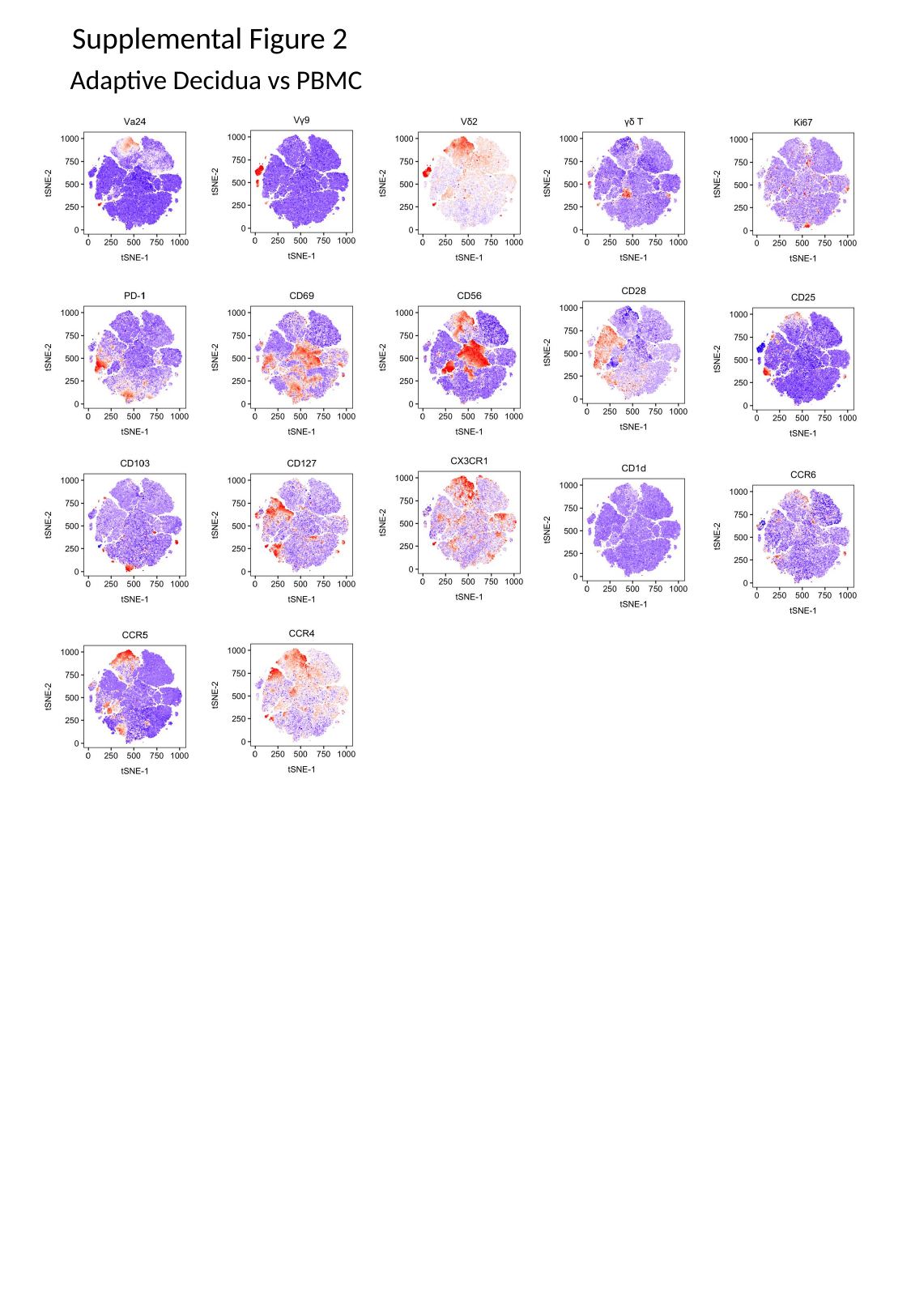

Supplemental Figure 2
Adaptive Decidua vs PBMC

## Slide 4
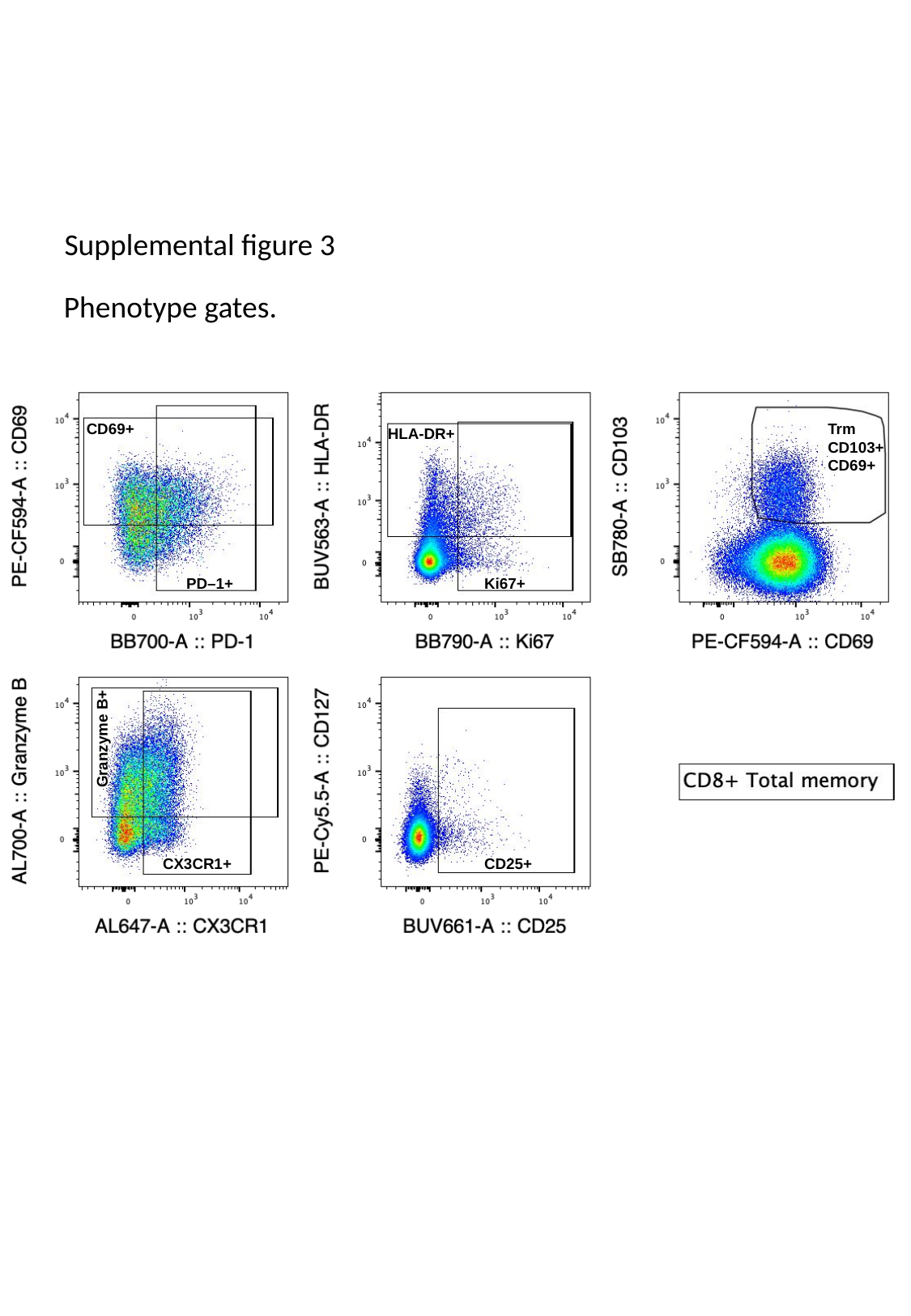

Supplemental figure 3
Phenotype gates.
CD69+
Trm
CD103+
CD69+
HLA-DR+
PD–1+
Ki67+
Granzyme B+
CX3CR1+
CD25+

## Slide 5
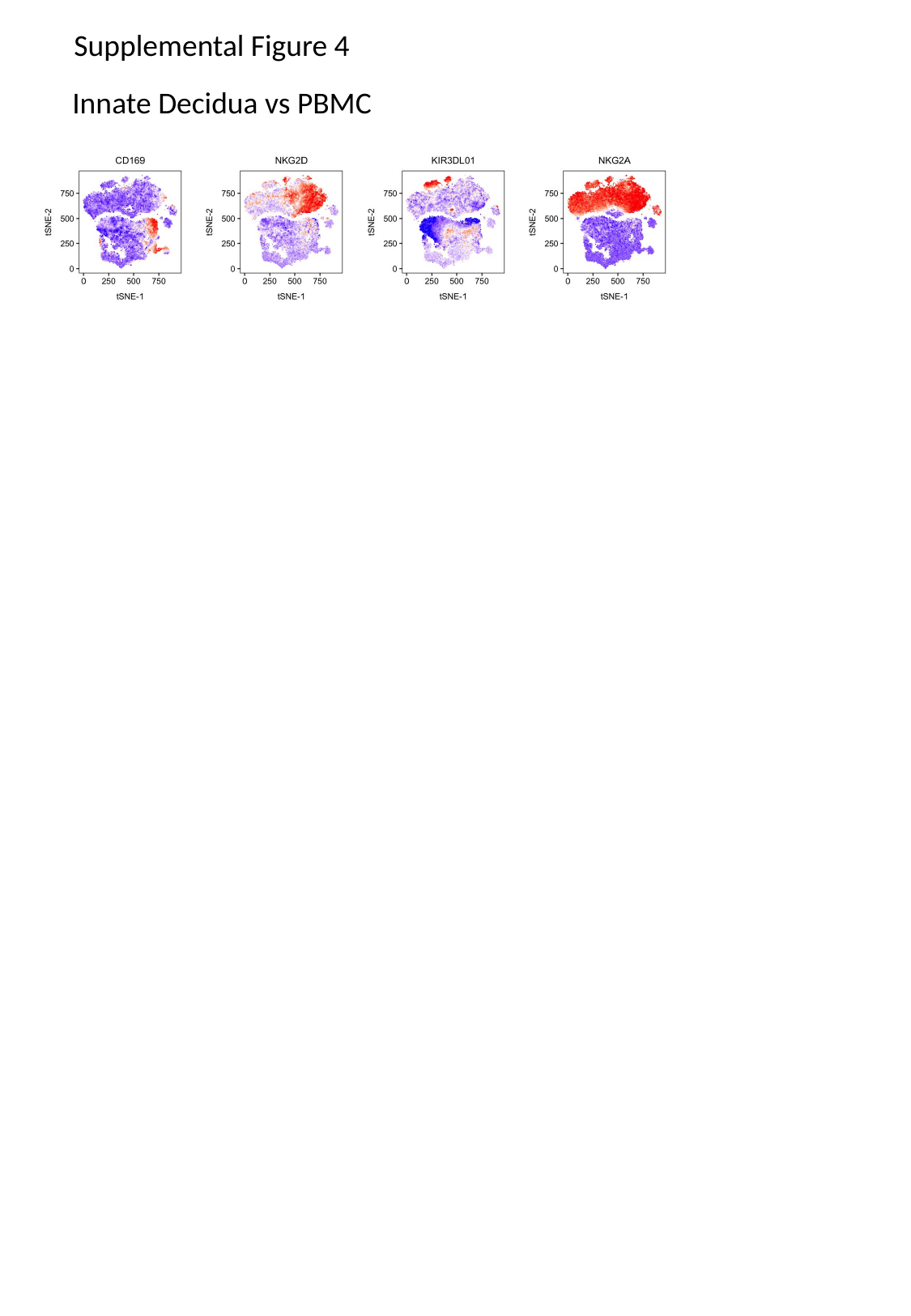

Supplemental Figure 4
Innate Decidua vs PBMC

## Slide 6
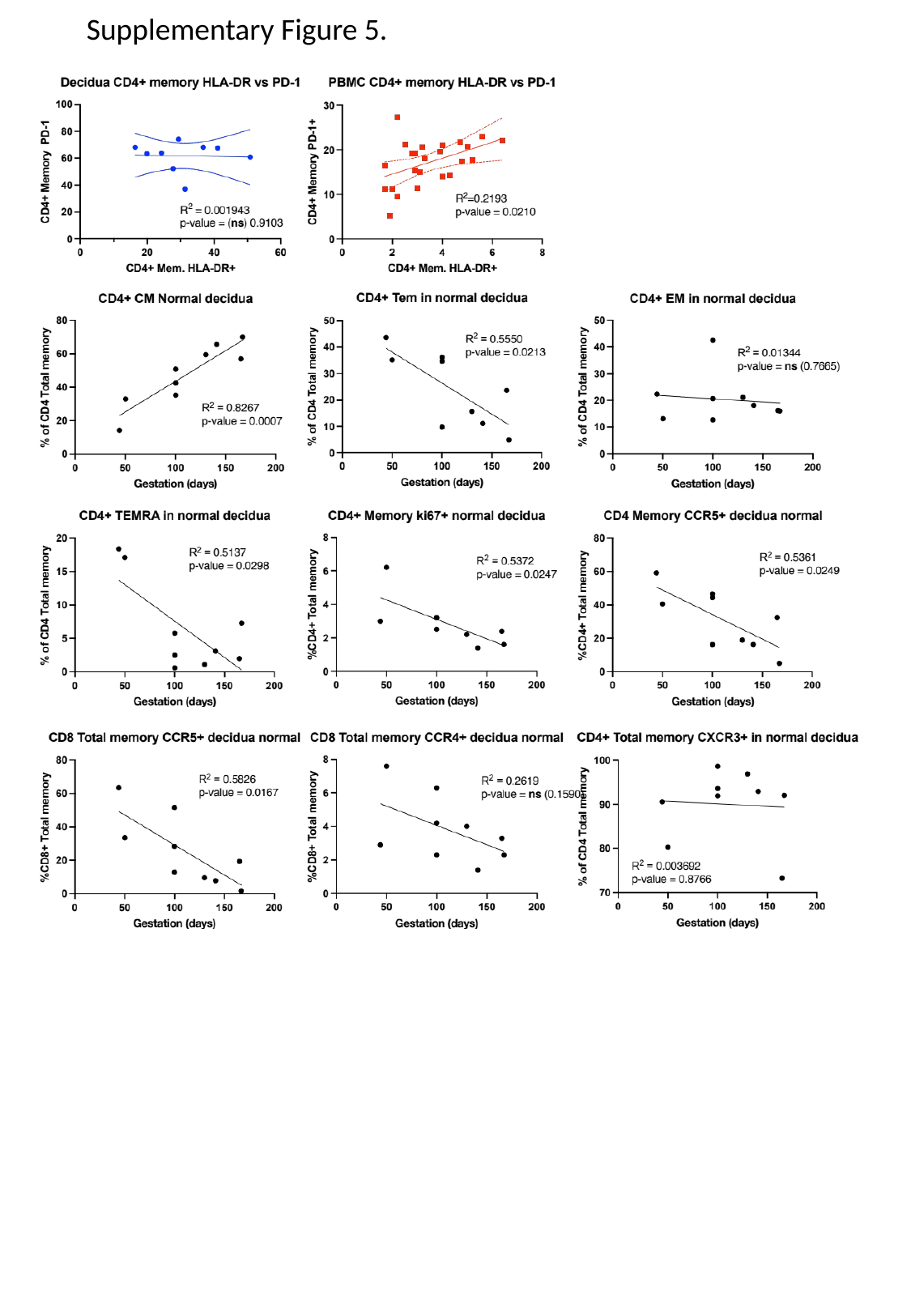

Supplementary Figure 5.

## Slide 7
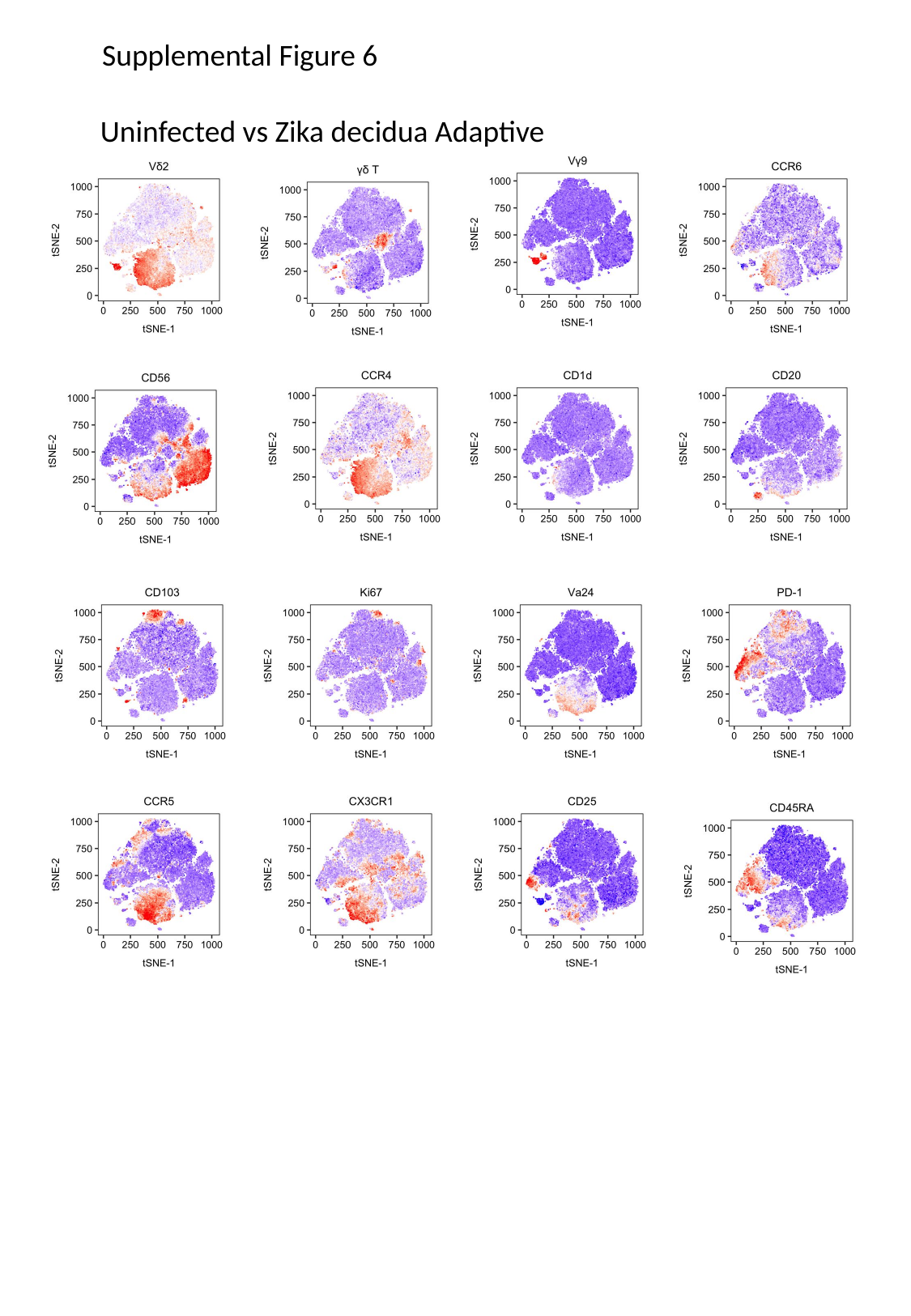

Supplemental Figure 6
Uninfected vs Zika decidua Adaptive

## Slide 8
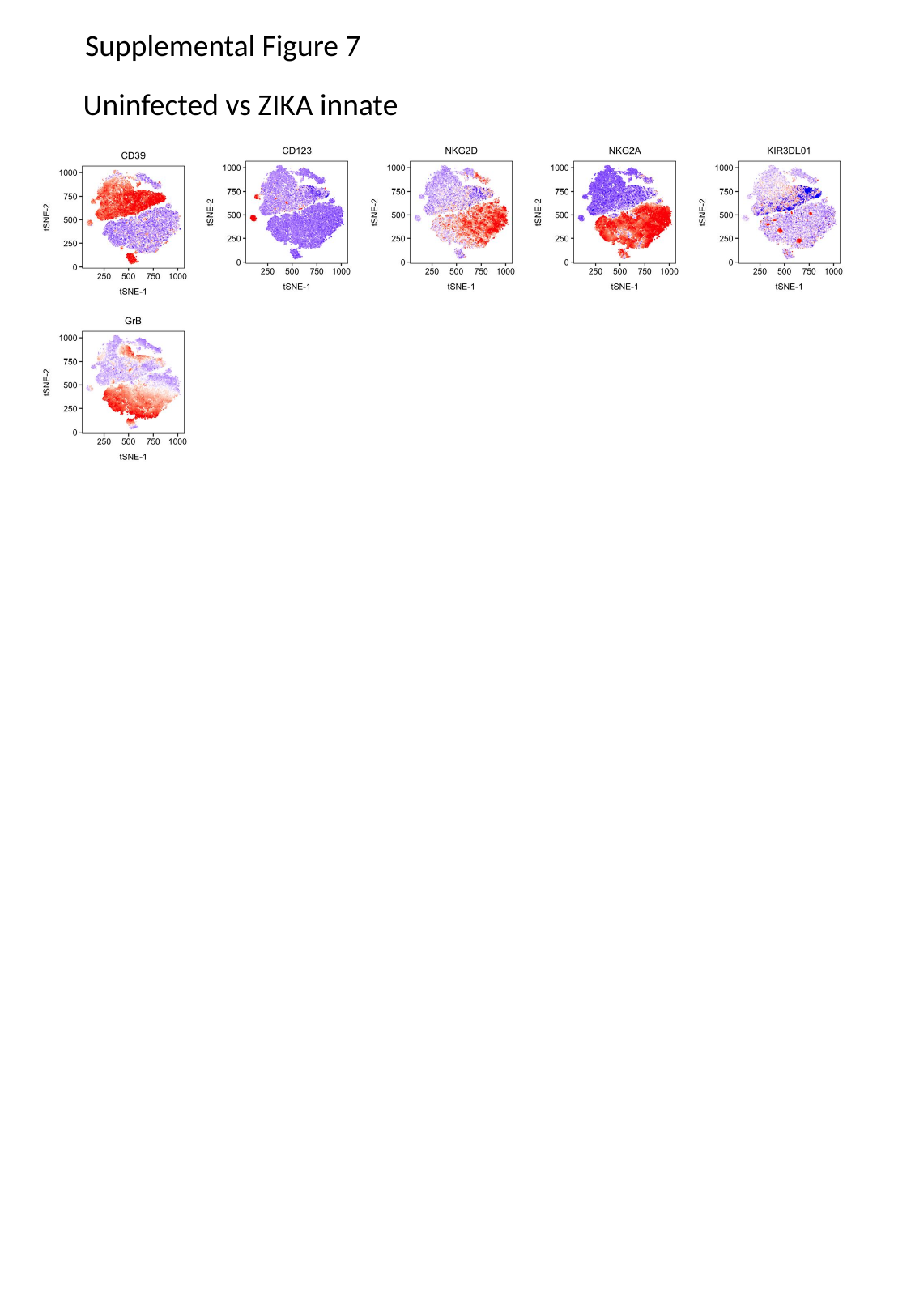

Supplemental Figure 7
Uninfected vs ZIKA innate
