# Supplementary material for: Immune Profile of the Normal Maternal-Fetal Interface in Rhesus Macaques and Its Alteration Following Zika Virus Infection
Source: Front Immunol. 2021 Jul 29;12:719810. doi: 10.3389/fimmu.2021.719810 (PMC8358803; doi:10.3389/fimmu.2021.719810)
Supplement: Supplementary file 2 [file Presentation_2.pptx]

## Slide 1
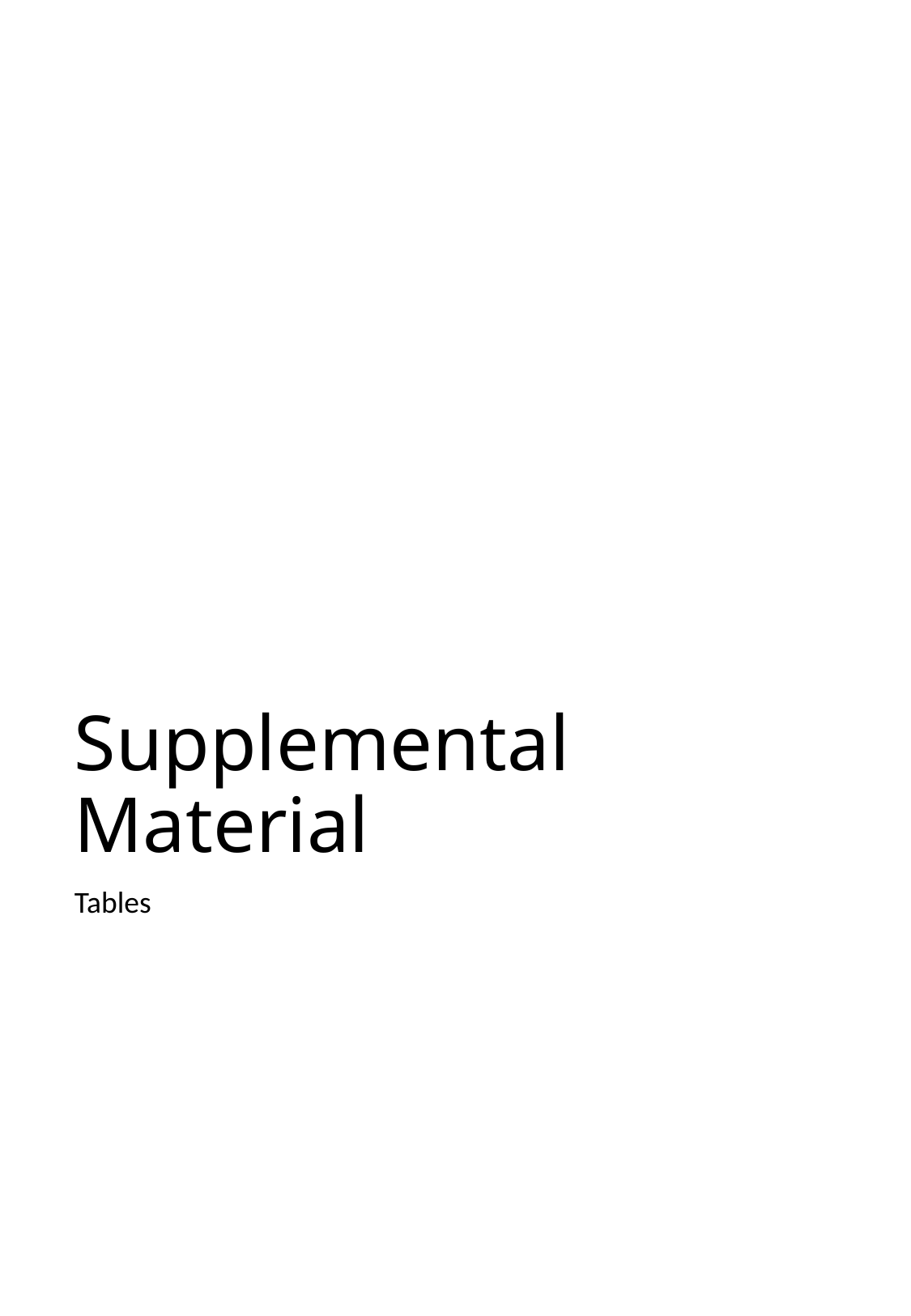

# Supplemental Material
Tables

## Slide 2
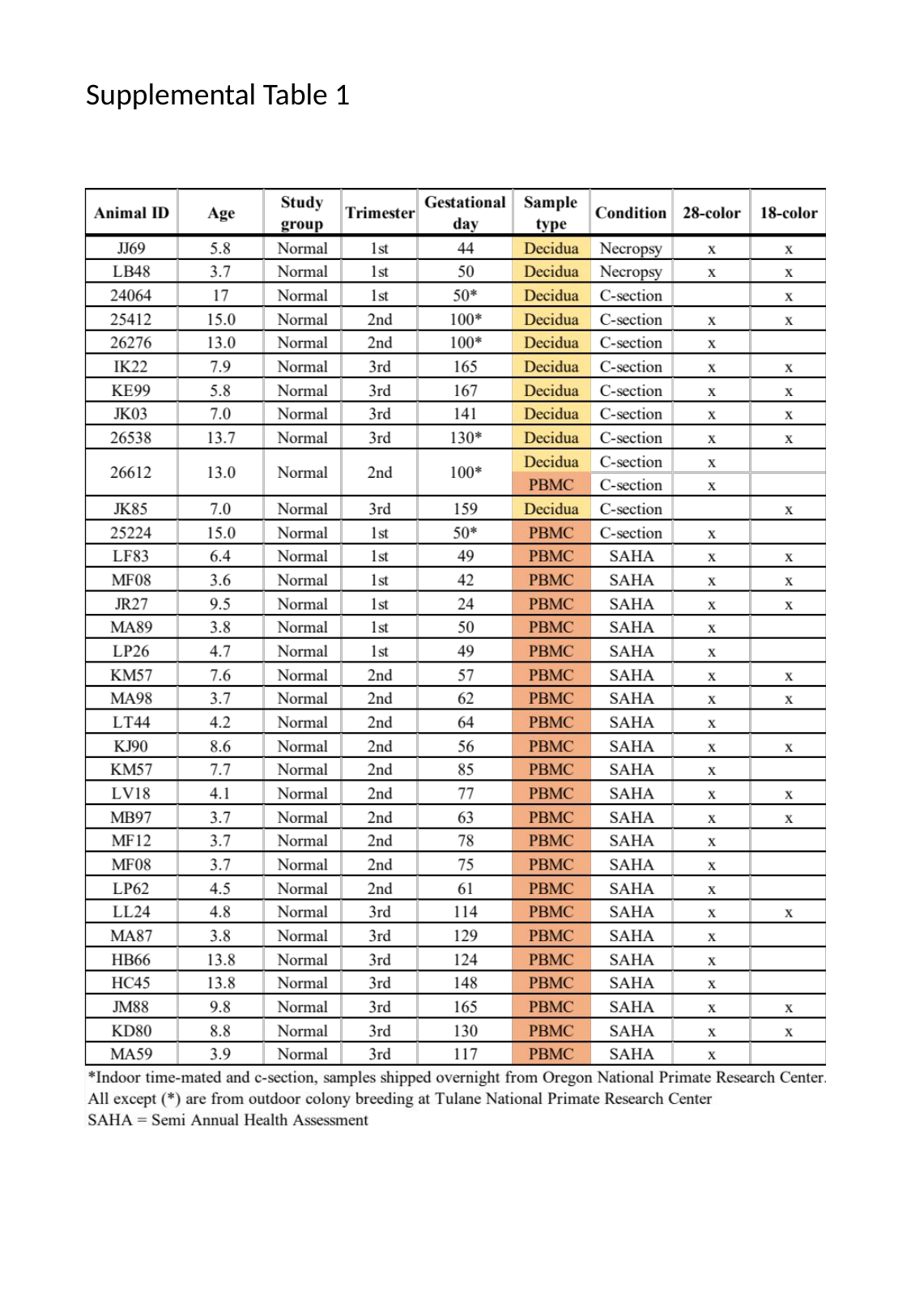

Supplemental Table 1

## Slide 3
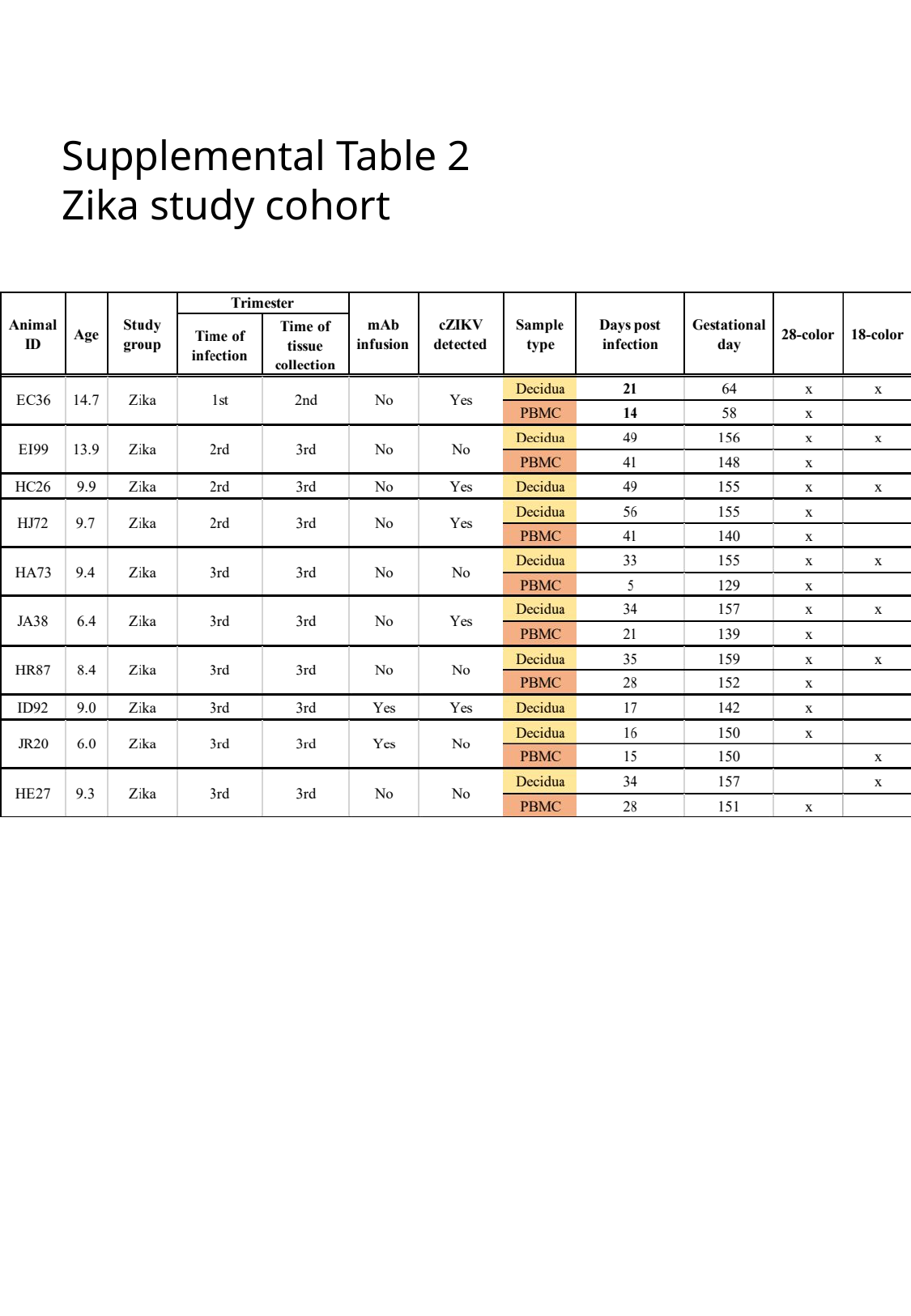

Supplemental Table 2
Zika study cohort

## Slide 4
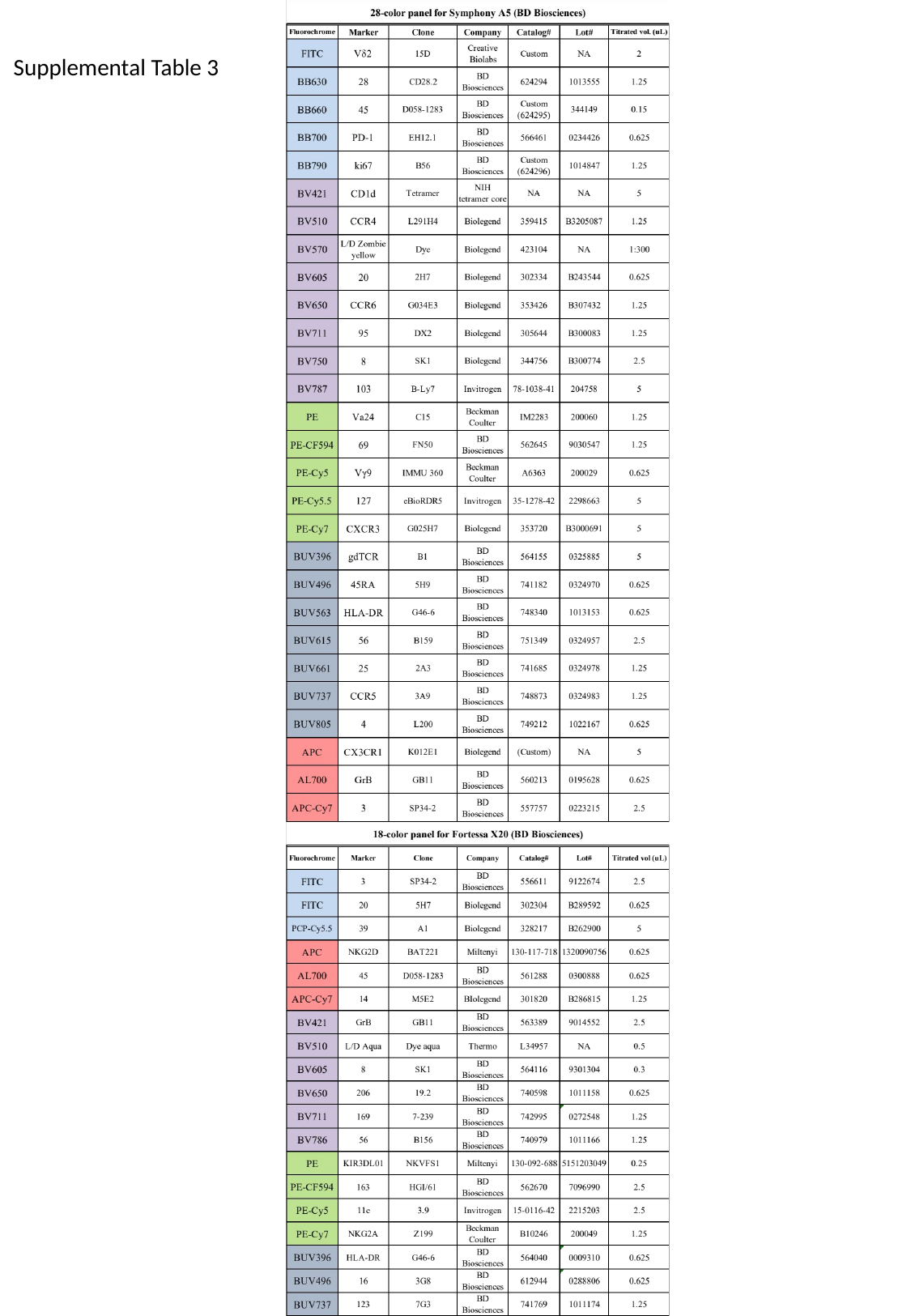

Supplemental Table 3

## Slide 5
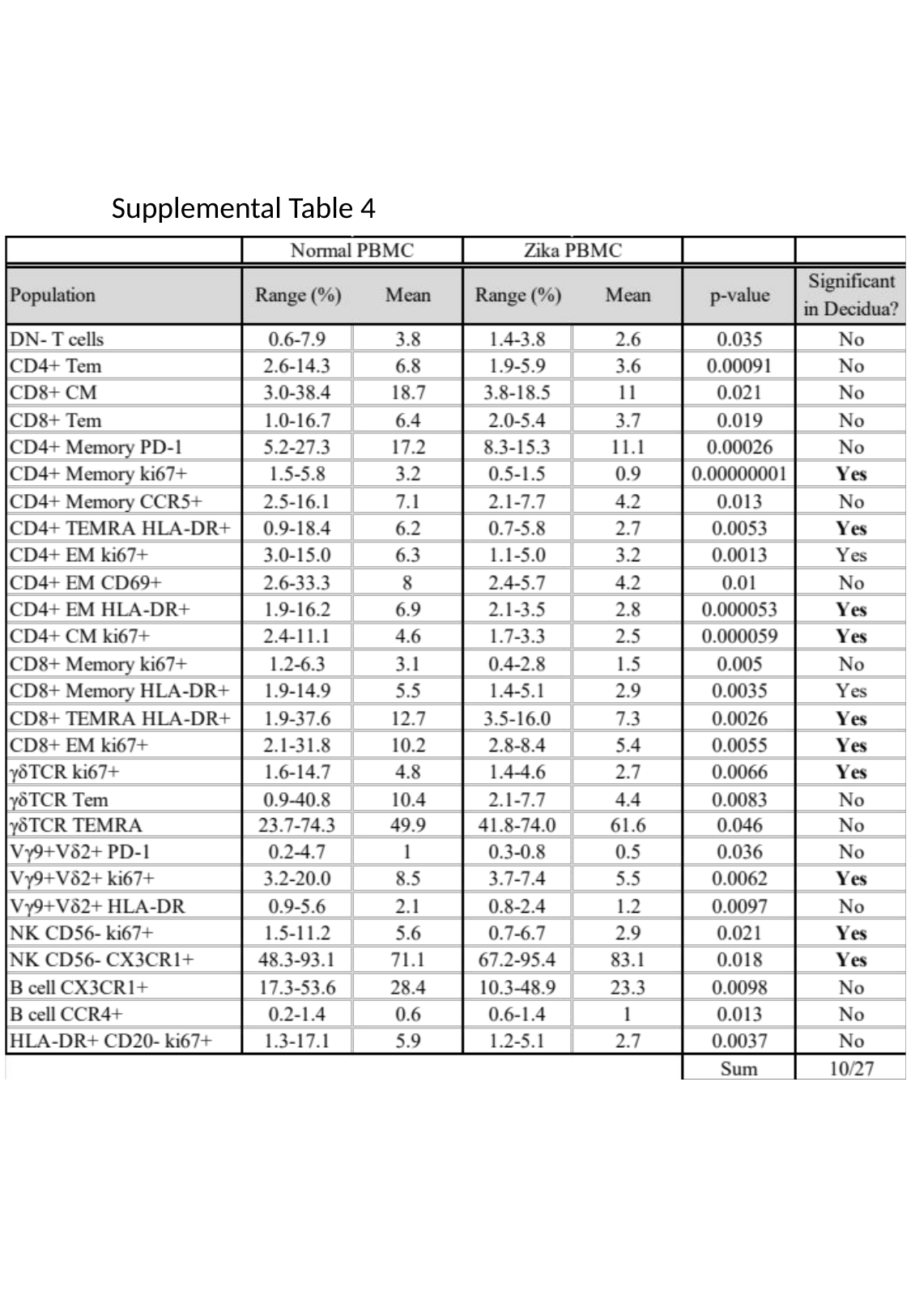

Supplemental Table 4
